# Transcriptome alterations in peripheral blood B cells of patients with multiple sclerosis receiving immune reconstitution therapy

**DOI:** 10.1186/s12974-023-02859-x

**Published:** 2023-08-02

**Authors:** Michael Hecker, Brit Fitzner, Nina Boxberger, Elena Putscher, Robby Engelmann, Wendy Bergmann, Michael Müller, Isis Ludwig-Portugall, Margit Schwartz, Stefanie Meister, Ales Dudesek, Alexander Winkelmann, Dirk Koczan, Uwe Klaus Zettl

**Affiliations:** 1 Division of Neuroimmunology, Department of Neurology, Rostock University Medical Center, Gehlsheimer Str. 20, 18147 Rostock, Germany; 2 Clinic III (Hematology, Oncology and Palliative Medicine), Special Hematology Laboratory, Rostock University Medical Center, Ernst-Heydemann-Str. 6, 18057 Rostock, Germany; 3 Core Facility for Cell Sorting and Cell Analysis, Rostock University Medical Center, Schillingallee 70, 18057 Rostock, Germany; 4 Miltenyi Biotec B.V. & Co. KG, Robert-Koch-Str. 1, 17166 Teterow, Germany; 5 Institute of Immunology, Rostock University Medical Center, Schillingallee 70, 18057 Rostock, Germany

**Keywords:** Multiple sclerosis, Immune reconstitution therapy, Peripheral blood, B cells, Transcriptome profiling, Gene interaction network analysis, Biomarker identification

## Abstract

**Background:**

Multiple sclerosis (MS) is a chronic, inflammatory and neurodegenerative disease that leads to irreversible damage to the brain and spinal cord. The goal of so-called "immune reconstitution therapies" (IRTs) is to achieve long-term disease remission by eliminating a pathogenic immune repertoire through intense short-term immune cell depletion. B cells are major targets for effective immunotherapy in MS.

**Objectives:**

The aim of this study was to analyze the gene expression pattern of B cells before and during IRT (i.e., before B-cell depletion and after B-cell repopulation) to better understand the therapeutic effects and to identify biomarker candidates of the clinical response to therapy.

**Methods:**

B cells were obtained from blood samples of patients with relapsing–remitting MS (*n* = 50), patients with primary progressive MS (*n* = 13) as well as healthy controls (*n* = 28). The patients with relapsing MS received either monthly infusions of natalizumab (*n* = 29) or a pulsed IRT with alemtuzumab (*n* = 15) or cladribine (*n* = 6). B-cell subpopulation frequencies were determined by flow cytometry, and transcriptome profiling was performed using Clariom D arrays. Differentially expressed genes (DEGs) between the patient groups and controls were examined with regard to their functions and interactions. We also tested for differences in gene expression between patients with and without relapse following alemtuzumab administration.

**Results:**

Patients treated with alemtuzumab or cladribine showed on average a > 20% lower proportion of memory B cells as compared to before IRT. This was paralleled by profound transcriptome shifts, with > 6000 significant DEGs after adjustment for multiple comparisons. The top DEGs were found to regulate apoptosis, cell adhesion and RNA processing, and the most highly connected nodes in the network of encoded proteins were ESR2, PHB and RC3H1. Higher mRNA levels of *BCL2*, *IL13RA1* and *SLC38A11* were seen in patients with relapse despite IRT, though these differences did not pass the false discovery rate correction.

**Conclusions:**

We show that B cells circulating in the blood of patients with MS undergoing IRT present a distinct gene expression signature, and we delineated the associated biological processes and gene interactions. Moreover, we identified genes whose expression may be an indicator of relapse risk, but further studies are needed to verify their potential value as biomarkers.

**Supplementary Information:**

The online version contains supplementary material available at 10.1186/s12974-023-02859-x.

## Background

Multiple sclerosis (MS) is a chronic immune-mediated central nervous system (CNS) disorder characterized by inflammatory demyelination, neuro-axonal degeneration and reactive astrogliosis [1]. MS affects approximately 2.8 million people worldwide, with a ~ 3 times higher prevalence in women compared to men [2]. The disease is typically diagnosed in adults aged 20–40 years. The diagnosis of MS relies on the integration of clinical, imaging and laboratory findings [3]. Disease activity and progression are defined by reversible episodes of new or worsening neurological deficits (also known as relapses), lesion activity in magnetic resonance imaging (MRI) and accumulation of disability over time. The spectrum of MS phenotypes is categorized into relapsing–remitting MS (RRMS), primary progressive MS (PPMS) and secondary progressive (SPMS) [4]. In most patients (~ 85–90%), acute relapses and periods of stability characterize the first years of the disease (RRMS) before a gradual worsening of clinical disability becomes prominent (SPMS). A minority of patients (~ 10–15%) have a progressive disease course from onset (PPMS). The etiology of MS remains unclear, but various genetic, environmental and lifestyle factors are known to contribute to disease development and severity [5, 6].

No curative treatment is yet available for MS. Disease-modifying therapies (DMTs) for MS, especially for RRMS, can favorably change the quality of life and long-term outlook for many patients [7]. They act by suppression or modulation of immune function to reduce the rate and severity of relapses, prevent lesion formation and delay the accumulation of permanent disability [8, 9]. DMTs for MS can be categorized into ongoing or maintenance therapies that are continuously administered (e.g., fingolimod, glatiramer acetate and natalizumab) and newer so-called pulsed immune reconstitution therapies (IRTs) that are administered in short courses and have the potential to induce long-term drug-free disease remission [10, 11]. The concept of IRTs is to eliminate a pathogenic adaptive immune repertoire through intense short-term immune cell depletion and then allow the immune system to renew itself. Examples of pulsed IRTs for MS are alemtuzumab and cladribine. Alemtuzumab is a humanized monoclonal antibody against CD52, which is highly expressed on B and T cells [12–14]. The therapy with alemtuzumab leads to a rapid and long-lasting depletion of CD52^+^ cells, followed by a slow repopulation arising from hematopoietic precursor cells. Alemtuzumab is infused for 5 consecutive days in the first course and for 3 days in the second course 1 year later, though up to 2 additional treatment courses may be considered as needed. Cladribine is a chlorinated analogue of deoxyadenosine that is activated through phosphorylation preferentially in lymphocytes. Activated cladribine interferes with DNA synthesis and repair and triggers apoptosis [15, 16]. Cladribine tablets are administered over 4–5 consecutive days at months 0 and 1 (first year of treatment) and at months 12 and 13 (second year of treatment). Ocrelizumab, an anti-CD20 monoclonal antibody, is currently the only approved DMT for PPMS [17, 18]. As the therapeutic options in PPMS are limited, repeated pulse therapy with corticosteroids has occasionally been used [19, 20]. However, patient selection, risk stratification and therapy guidance are challenging in the context of IRTs. Thus, deeper insights into their mechanisms of action and reliable markers to identify patients with suboptimal treatment response and to inform physicians whether to retreat or to switch therapy are urgently needed.

B cells play a key role in the pathobiology of MS, which is underscored by the high efficacy of therapeutic strategies that target these cells [7, 8] and the fact that they are a primary target of Epstein–Barr virus (EBV) infection, the leading risk factor for MS [21–23]. B cells are thought to contribute to MS through their antigen-presenting function and the formation of tertiary lymphoid-like structures in the CNS, which are the likely source of an abnormal immunoglobulin production detectable in the cerebrospinal fluid (CSF) [24, 25]. Circulating B cells from individuals with MS are potent activators of autoreactive T cells as they exhibit an imbalance in the secretion of pro- and anti-inflammatory cytokines and express increased levels of co-stimulatory molecules, such as CD80 [26, 27]. Within the B-cell population, memory B cells may be a driver subset in MS, as indicated by genetic studies [28] and studies of cell population shifts in the peripheral blood in response to DMTs [29]. The therapy with alemtuzumab leads to an effective depletion of circulating CD19^+^ B cells by ~ 90%, which is followed by repopulation, with total B-cell counts returning to baseline at 3 months and then rising further to ~ 165% of baseline at 12 months after treatment [30]. However, the apparent increase in the number of CD19^+^ B cells is generated by newly produced immature (transitional) B cells and mature naive B cells, whereas CD27^+^ memory B-cell recovery is slow, reaching only ~ 25% of baseline by month 12 [30, 31]. Similarly, cladribine induces a 70–90% depletion of B cells [32]. Within 1 year after the first administration of cladribine (i.e., until the second treatment course), the B cells repopulate to 70–80% of baseline, but the number of memory B cells remains persistently low for over 12 months (~ 80% reduction compared to baseline) [33–36]. These changes in the immune reconstitution phase are associated with a decrease in pro-inflammatory responses [11, 37]. However, previous studies could not detect significant differences in the recovery of any blood cell population between patients with and without recurrent disease activity after treatment [32, 38–41]. Moreover, the effects of these IRTs for MS on the transcriptome profile of B cells have not been investigated so far.

In this study, we analyzed B cells from the peripheral blood of MS patients and healthy controls at the cellular and transcriptome level. We show that the gene expression pattern of B cells is substantially altered in patients receiving alemtuzumab or cladribine, which reflects the shift in B-cell subsets in response to these IRTs and affects a wide variety of biological functions. We also compared the transcriptome data between patients remaining free of relapses and patients who developed a relapse in the year following alemtuzumab administration to find potential biomarkers of treatment outcome. For selected genes, the possible involvement in immune mechanisms related to MS will be discussed.

## Methods

### Study groups

The study population comprised 63 patients with MS and 28 healthy controls aged over 18 years. In this cohort, we have already investigated the effects of MS-associated genetic variants on the processing of RNAs, as described in detail in our previous publications [42, 43]. Patient care and treatment followed routine clinical practice at the Rostock University Medical Center. In brief, the diagnosis of MS was confirmed based on the revised McDonald criteria [3]. The patients were monitored for the occurrence of relapses, and their degree of neurological disability was rated with the Expanded Disability Status Scale (EDSS) [44]. The therapeutic management of the patients was carried out according to the guidelines of the German Society of Neurology and the approved product labels. The study was conducted with approval by the local ethics committee of the University of Rostock and in compliance with the principles of the current Declaration of Helsinki.

**Fig. 1:**
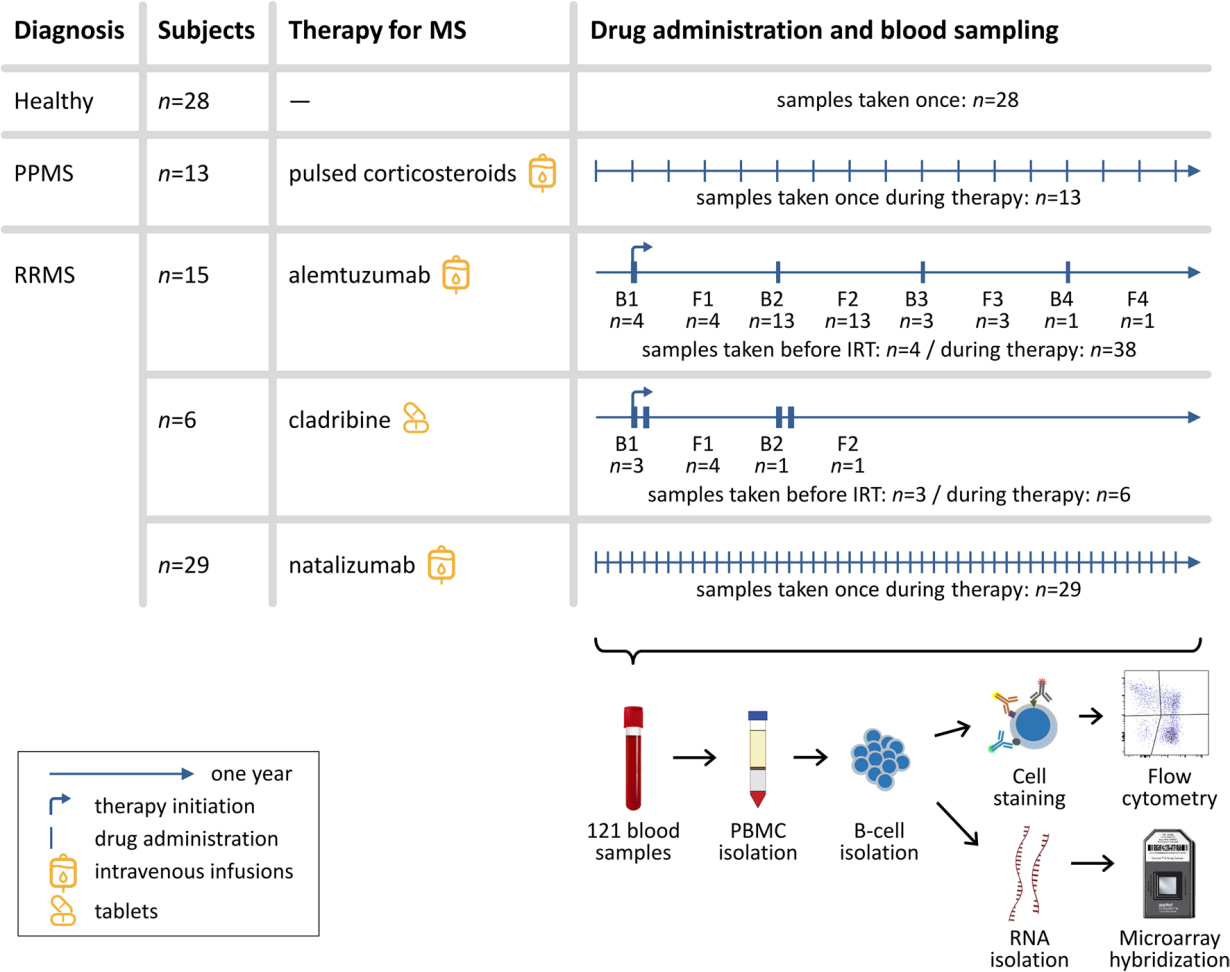
Overview of the study. Peripheral blood was taken from healthy controls, patients with primary progressive MS (PPMS) and patients with relapsing–remitting multiple sclerosis (RRMS). The patients with RRMS received either monthly infusions of natalizumab (*n* = 29) or a pulsed immune reconstitution therapy (IRT) with alemtuzumab (*n* = 15) or cladribine (*n* = 6). The timelines of drug administration are shown in blue. For the study groups healthy, PPMS and natalizumab, we collected one sample per individual, whereas in the case of IRTs, up to four samples were collected per patient before (B) and/or following (F) an annual treatment course. In total, we collected 121 blood samples from 91 subjects. We then isolated peripheral blood mononuclear cells (PBMC) before isolating untouched B cells by magnetic separation. A fraction of the B cells was cryopreserved and later used for B-cell phenotyping by multicolor flow cytometry. From the remaining B cells, total RNA was extracted, labeled and hybridized to high-density Clariom D arrays to obtain transcriptome profiles

An overview on the treatment of the patients and the blood sampling is shown in Fig. 1. The patients with PPMS (*n* = 13) received intravenous methylprednisolone for 3–5 days every 3–6 months since at least 3 years. A subgroup of the patients with RRMS (*n* = 29) was treated with monthly infusions of natalizumab for a minimum of 1 year. From the patients in these two study groups, a single blood sample was collected just before an upcoming infusion. The other patients with RRMS received an IRT with either intravenous alemtuzumab (*n* = 15) or oral cladribine (*n* = 6). From these patients, up to four blood samples were collected immediately before (B) and/or following (F) the 1st, 2nd, 3rd or 4th annual treatment course. The F samples were taken during a regular clinical visit usually 6–9 months after the start of the previous treatment course. From alemtuzumab-treated patients, the B samples and F samples were always obtained in a paired manner (i.e., before B-cell depletion and after B-cell repopulation). This was not the case for the cladribine-treated patients. From 7 patients, a blood sample was also taken before the initiation of the IRT. These B1 samples are hereinafter referred to as the "before IRT" group.

### Blood sample processing

In total, 121 peripheral blood samples were collected with prior written informed consent from each participant. The blood (~ 20 ml) was drawn into tubes with ethylenediaminetetraacetic acid (EDTA). Immediately following the blood withdrawal, peripheral blood mononuclear cells (PBMC) were isolated using Ficoll density gradient separation (Histopaque-1077, Sigma-Aldrich). Untouched B cells were then enriched from the PBMC by negative selection using the Pan B Cell Isolation Kit (Miltenyi Biotec). The number of freshly isolated cells was counted under a microscope, and the purity of B cells was assessed as described previously [42]. From each sample, 200,000 B cells were cryopreserved in freezing medium (Biofreeze, Biochrom) in the vapor phase of liquid nitrogen at below − 140 °C until performing the flow cytometry analysis. The remaining B cells were lysed in QIAzol Lysis Reagent (Qiagen) and stored at − 80 °C. Total RNA was later isolated using the miRNeasy Mini Kit (Qiagen) with the RNase-free DNase set (Qiagen). RNA concentrations were quantified using a NanoDrop ND-1000 Spectrophotometer (Thermo Fisher Scientific). The integrity of the RNA samples was checked using an Agilent 2100 Bioanalyzer with RNA 6000 Pico kits (Agilent Technologies).

### Flow cytometry

The frequencies of B-cell subpopulations were analyzed using a BD FACSAria IIIu system following the guidelines for the use of flow cytometry in immunological studies [45]. As described earlier [42], all the cryopreserved B cells were quickly thawed in a 37 °C water bath, washed with phosphate-buffered saline and then stained with the fluorochrome-conjugated antibodies CD19-PerCP, CD23-BV421, CD27-PE, CD38-PE/Dazzle594 (all from BioLegend), CD21-APC, CD24-APC-Vio770 (Miltenyi Biotec) and IgD-BV750 (BD). Zombie Green dye was used for live/dead staining of the cells (BioLegend). FcR blocking reagent (Miltenyi Biotec) was used to block unspecific binding of antibodies to Fc receptors. Data acquisition and compensation calculations were performed using the BD FACSDiva software version 8.0.2.

The obtained data were processed in the FlowJo software version 10 (BD) for determining the percentages of B-cell subsets [45–48] with slight modifications compared to our previous study [42]. First, outlier events were removed using the FlowAI plugin version 2.1 [49]. Live single CD19^+^ cells were then identified based on their forward and side scatter properties and the signals for the live/dead and CD19 markers. Finally, we gated on CD27^−^IgD^+^ naive B cells, CD27^+^IgD^+^ non-switched memory B cells, CD27^+^IgD^−^ switched memory B cells, CD27^−^IgD^−^ memory B cells, CD27^++^CD38^++^ plasmablasts, CD24^++^CD38^++^ transitional B cells [46], CD21^−/low^CD38^−/low^ B cells [47] and CD23^high^ B cells [48] (Additional file 1: Fig. S1).

The data were visualized in box/beeswarm plots using the R package beeswarm. Pairwise comparisons between the study groups were performed with Tukey post hoc tests for linear mixed-effects models (LMM) using the R package multcomp [50]. The subjects were treated as a random effect in the LMMs to consider for repeated measurements.

### Transcriptome profiling

The gene expression levels in B cells were measured by high-density Clariom D arrays for human (Applied Biosystems), which contain > 6.7 million 25mer oligonucleotide probes. For this purpose, amplified, fragmented and biotinylated single-stranded sense strand DNA was generated from 100 ng of total RNA per sample using the GeneChip WT PLUS Reagent Kit (Applied Biosystems). The hybridization of the arrays was conducted for 16 h at 45 °C in a GeneChip Hybridization Oven 645 (Affymetrix). After washing and staining in a GeneChip Fluidics Station 450 (Affymetrix), the arrays were scanned using a GeneChip Scanner 3000 7G (Affymetrix). The scans were processed with the Affymetrix GeneChip Command Console version 4.0 to extract signal intensities. Data normalization, probe set summarization and log2 transformation were finally performed with the Transcriptome Analysis Console version 4.0.2 (Applied Biosystems) by applying the signal space transformation robust multi-array average algorithm.

### Identification of differentially expressed genes

We screened for differentially expressed genes (DEGs) between the 6 study groups (healthy, PPMS, before IRT, alemtuzumab, cladribine and natalizumab) in the R software environment for statistical computing. This was done by fitting LMMs with a random effect for each subject to the data for each gene level probe set (also referred to as transcript cluster or simply "gene" in the following) using the R package lme4 [51]. Type II Wald χ^2^ tests were then calculated for the models with the R package car [52] to obtain *p* values, which were adjusted for multiple testing using the false discovery rate (FDR) approach [53]. The significance level was generally set to 5%.

For the subgroup of patients who received alemtuzumab, we have also specifically analyzed the gene expression changes in response to each annual treatment course. For this purpose, the respective data for B samples and F samples were compared with paired *t* tests and used to calculate log2 fold changes (log2FC). Genes with strong changes in expression in the enriched B cells were filtered by requesting a *p *value < 0.05 and a log2FC of <  − 1 or > 1, which means that the transcript level is on average reduced by > 50% or increased by > 100% at the follow-up timepoint as compared to before the treatment course. A log2FC of even <  − 3 or > 3 (i.e., a mean decrease by > 87.5% or a mean increase by > 700%) was regarded as an extreme shift in expression.

We restricted the transcriptome analyses to transcript clusters with annotated Entrez Gene identifier. Moreover, we excluded genes that were not expressed in the B cells by eliminating probe sets with a log2 signal intensity < 4 in all samples.

### Clustering of genes

A hierarchical clustering of the top 500 DEGs across the 6 study groups (according to the Wald test *p* values) was performed on the basis of the complete linkage method and Pearson’s correlation coefficient as a measure of similarity. Based on this, the DEGs were grouped into clusters with distinct gene expression patterns. The signal intensities for each gene were centered and scaled (yielding z-scores) for visualization in a heatmap. Moreover, a line chart was drawn for each cluster by connecting for each gene the average standardized expression level of the healthy controls and each subgroup of patients with MS.

### Analysis of gene expression in B-cell subsets

Differences in the B-cell transcriptome signature between the study groups are presumably related to differences in the composition of B-cell subpopulations. We have, therefore, taken a closer look on the expression of the top 500 DEGs in B-cell subsets using the RNA sequencing data set from Monaco et al. [54]. These data provide median transcripts per million values for 29 sorted human immune cell types, including CD27^−^IgD^+^ naive B cells, CD27^+^IgD^+^ non-switched memory B cells, CD27^+^IgD^−^CD38^low^ switched memory B cells, CD27^−^IgD^−^ exhausted memory B cells and CD27^+^IgD^−^CD38^high^ plasmablasts of healthy donors. We used the data to derive z-scores, which were averaged over all genes in a cluster as a measure of its cell type specificity.

### Mapping of genes to biological processes

To assign the top 500 DEGs to functional categories, we utilized the biological process terms of the Gene Ontology (GO) annotation. Overrepresented GO terms were identified for each gene cluster using the R packages org.Hs.eg.db and GOstats [55]. Accordingly, hypergeometric tests were performed to filter GO terms that are significantly enriched with *p* < 0.05 in the cluster gene sets as compared to the reference gene set (defined as all expressed genes with Entrez Gene identifier). The results were further narrowed down by excluding GO terms that contain > 5000 genes of the reference set or < 20% of the genes of a cluster set. Further information on individual genes were derived from PubMed and the GeneCards database [56].

### Gene interaction network analysis

We explored interactions between the genes with differential expression across the 6 study groups using the GeneMANIA plugin (version 3.5.2) [57] for the network visualization software Cytoscape (version 3.9.0) [58]. This was done by entering the gene symbols of the top 500 DEGs to search for interactions in the data set for homo sapiens with the default configuration. This yielded a network of (pairwise) physical interactions, which were retrieved from protein–protein interaction studies. The color and size of the network nodes (i.e., the gene products) were used to indicate the cluster membership and the number of adjacent edges (i.e., the interactions), respectively.

### Search for potential markers of relapse

We examined whether the transcript levels in the B cells were associated with the clinical response to the IRT with alemtuzumab. For this purpose, we compared the data between patients with relapse and patients without relapse in the 12 months after a course of infusions. This comparison was made for all transcript clusters and for the timepoints B1, F1, B2 and F2. In addition to the mean difference (MD) in expression between the patient groups at each timepoint, significance values were calculated by Welch *t* tests. Genes whose expression might be related to relapse risk were then filtered by selecting expressed genes with Entrez Gene identifier with *p* < 0.05 and MD <  − 1 or > 1. The latter means that the expression is at least twice as high in one of the two groups than in the other group, as the processed data are in log2 scale. For a more stringent selection, we demanded an MD <  − 1 or > 1 for both B1 and B2 or for both F1 and F2. Clinicodemographic data were tested for association with relapses using Welch's *t* test, Mann–Whitney *U* test and Fisher's exact test.

## Results

### Characterization of the study cohort

The study population comprised 91 subjects in total [42, 43]. The patients with PPMS were on average older (mean age ± standard deviation: 58.7 ± 9.8 years) than the patients with RRMS (36.1 ± 10.6 years) and the healthy controls (28.0 ± 8.9 years) at the timepoint of blood collection. There were also differences in the proportion of women (PPMS: 38.5%, RRMS: 66.0%, healthy: 60.7%). The MS patients had a mean disease duration of 8.2 ± 6.6 years and a mean EDSS score of 3.0 ± 1.6 (10 patients with missing value). Four RRMS subgroups were defined by the current treatment (natalizumab, alemtuzumab, cladribine or before IRT), resulting in 6 study groups in total. The 7 patients in the before IRT group were previously treated with fingolimod (*n* = 3), glatiramer acetate (*n* = 3) or interferon-β-1b (*n* = 1). Blood samples were taken from these patients immediately before they received the first treatment course of alemtuzumab (*n* = 4) or cladribine (*n* = 3), called B1 samples, as well as during therapy. Detailed clinicodemographic information are provided in Additional file 2.

### Differences in the proportions of B-cell subpopulations

**Fig. 2:**
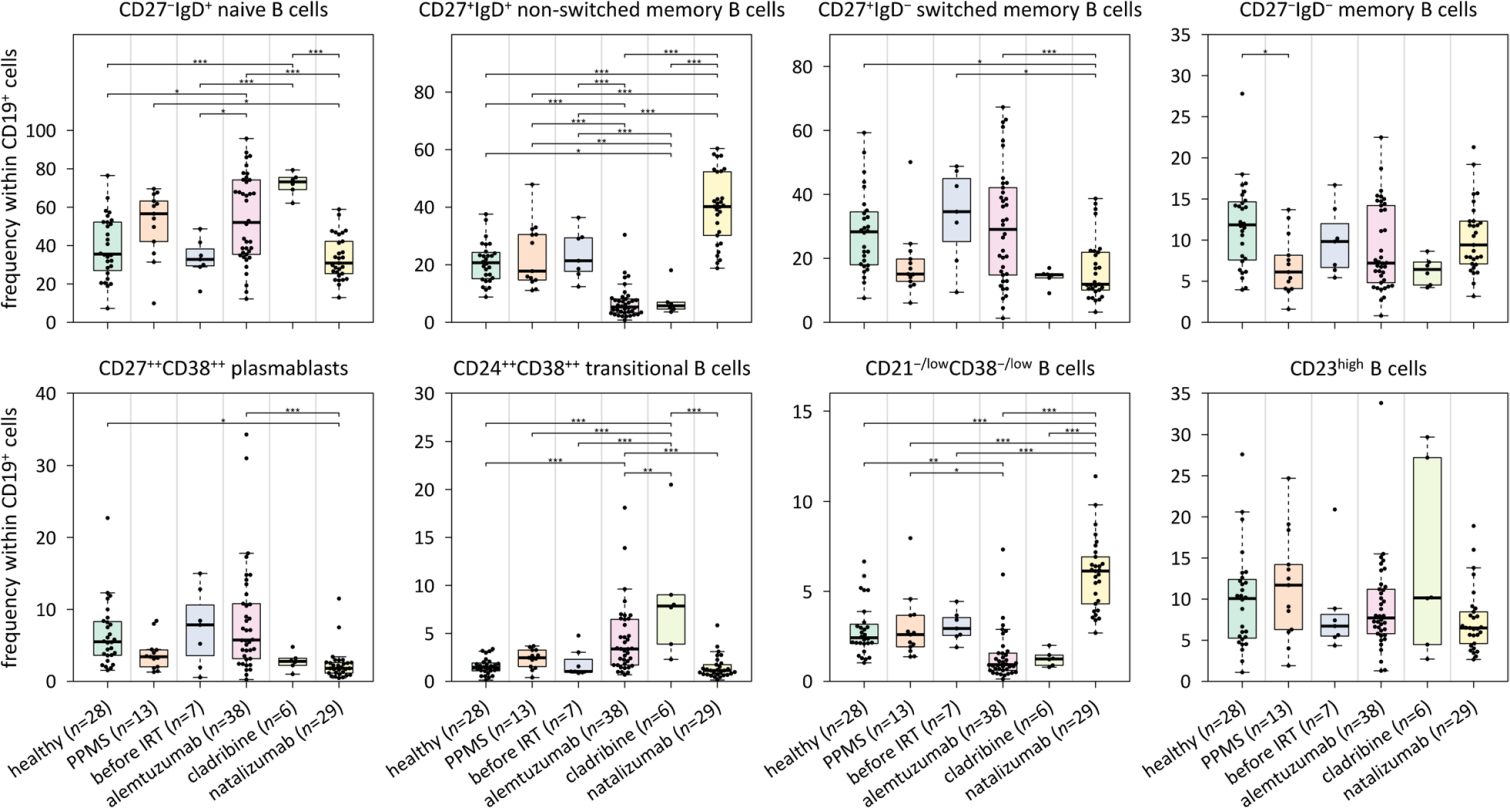
Frequencies of B-cell subpopulations across the study groups. Eight subsets of CD19^+^ cells were measured by flow cytometry as shown in Additional file 1: Fig. S1. In addition to the individual data points, the medians and interquartile ranges per group are depicted by box plots. The brackets indicate statistical significance in pairwise comparisons based on Tukey post hoc tests for linear mixed-effects models. The average percentages per group are given in Additional file 3. IRT = immune reconstitution therapy, PPMS = primary progressive multiple sclerosis, * *p* < 0.05, ** *p* < 0.01, *** *p* < 0.001

The enrichment of B cells from PBMC yielded an average number of 4.3 million cells at an average B-cell purity of 85.2% (3 samples with missing value) [42]. The samples were used to perform the B-cell phenotyping and the RNA analyses. With regard to the results from the flow cytometric measurements, the percentages of the different B-cell subsets across the 6 study groups are shown in Fig. 2 and tabulated in Additional file 3. Over all 121 samples, naive B cells constituted the most abundant subpopulation (45.1% of CD19^+^ cells on average), whereas transitional B cells and CD21^−/low^CD38^−/low^ B cells were relatively rare (2.9% and 3.1% on average, respectively). A mean proportion of 5.7% of the cells was identified as plasmablasts.

With the exception of CD23^high^ B cells, significant differences in the relative proportions were seen for all subpopulations when comparing the 6 study groups (Wald test *p* < 0.05). Particularly substantial shifts, remarkably in the opposite direction, were seen for the two IRT groups compared to the natalizumab group. For natalizumab-treated patients, we observed, on average, the highest percentages for non-switched memory B cells (39.7%) and CD21^−/low^CD38^−/low^ B cells (6.0%) but the lowest percentages for plasmablasts (2.3%) and transitional B cells (1.5%). In contrast, the samples obtained during IRT showed the lowest mean percentages for non-switched memory B cells (6.9% in the alemtuzumab group) and CD21^−/low^CD38^−/low^ B cells (1.3% in the cladribine group) but the highest mean percentages for plasmablasts (8.4% in the alemtuzumab group) and transitional B cells (8.6% in the cladribine group). These alterations at the cellular level are reflective of the very different mechanisms of actions of these treatments for RRMS. For a more detailed view, the changes in the composition of B cells during the course of therapy with alemtuzumab are visualized in Additional file 1: Fig. S2.

### Differences in the B-cell transcriptome profiles

**Fig. 3:**
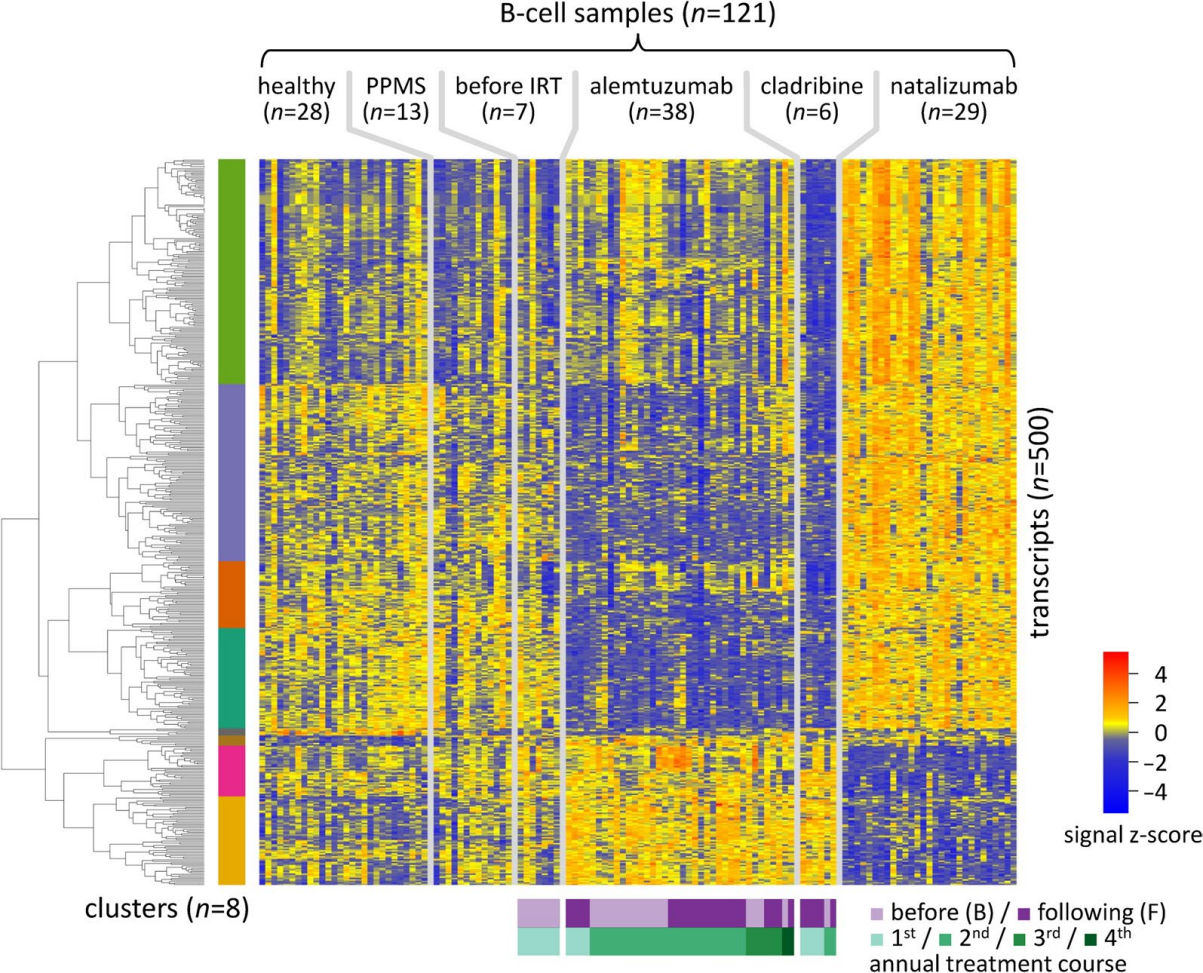
Heatmap of the top 500 differentially expressed genes. Differentially expressed genes (DEGs) between the 6 study groups were determined using linear mixed-effects models treating subjects as a random effect. For the top 500 DEGs (all with false discovery rate < 0.05), the scaled signal intensities (z-scores) are visualized, with blue indicating low expression and red indicating high expression. The genes (rows) were ordered by hierarchical clustering, and 8 gene clusters could be defined (shown by the colored bars on the left). The light and dark purple bars at the bottom indicate whether the blood samples were taken before or following the administration of alemtuzumab or cladribine. Paired B samples and F samples of an annual treatment course (represented by the green bars at the bottom) are always arranged in the same order. The samples and genes in the columns and rows are specified in Additional files 2 and 4, respectively. IRT = immune reconstitution therapy, PPMS = primary progressive multiple sclerosis

We next explored the differences at the transcriptome level between the study groups. The profiling with Clariom D arrays was based on a total of 135,750 gene level probe sets. The raw and processed data have been deposited in the Gene Expression Omnibus (GEO) database under accession number GSE190847 [43]. The assignment of the 121 samples to these data is given in Additional file 2. A subset of 21,587 transcript clusters were annotated with Entrez Gene identifiers and considered to be expressed in the B cells from the peripheral blood of the MS patients and/or healthy controls. The analysis for differential expression between the 6 study groups revealed strong transcriptome shifts. We could determine 6,280 DEGs using the threshold FDR = 0.05 (Additional file 4). The scaled signal intensities of the top 500 DEGs are visualized in Fig. 3.

**Fig. 4:**
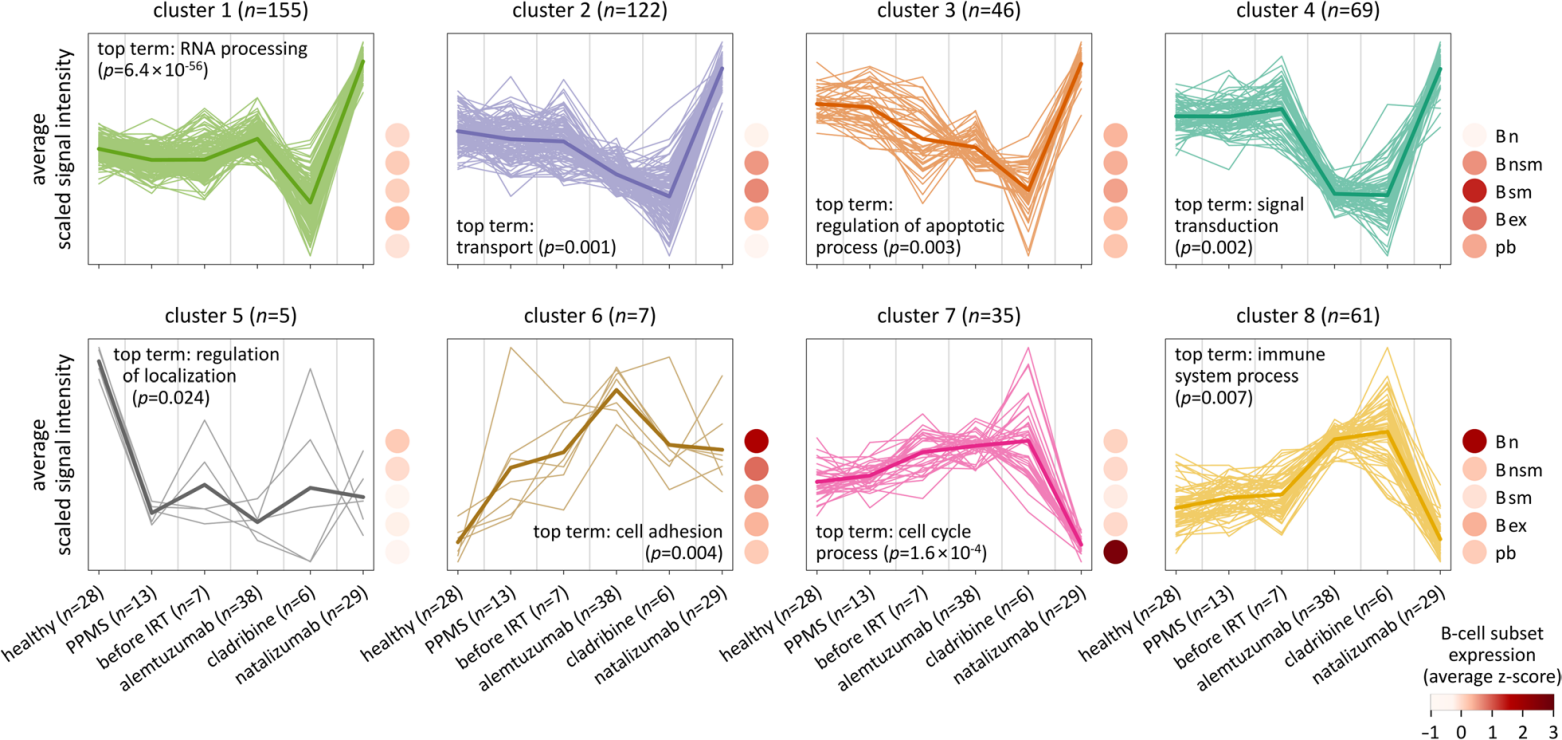
Gene expression patterns of the different clusters. B-cell transcriptome profiles were compared between healthy controls and subgroups of patients with multiple sclerosis. The top 500 differentially expressed genes (DEGs) were grouped in 8 clusters. In this plot, a line is drawn for each DEG by connecting the average standardized expression value of each study group. The cluster color code is as shown in Fig. 3. The thick line in each subpanel shows the mean of the means over all genes in the cluster. For each cluster, the most overrepresented gene functional term that is characteristic of the respective gene set is given. In addition, the dot plots on the right of each subpanel visualize the average expression of the genes in different B-cell subsets according to the data set by Monaco et al. [54]. The gene names are given in Additional file 4. B ex = exhausted memory B cells, B n = naive B cells, B nsm = non-switched memory B cells, B sm = switched memory B cells, IRT﻿ = immune reconstitution therapy, pb = plasmablasts, PPMS = primary progressive multiple sclerosis

The top 500 DEGs could be grouped into 8 clusters (Figs. 3, 4 and Additional file 4). Cluster 1 and cluster 2 comprised the largest numbers of genes (*n* = 155 and *n* = 122, respectively). The genes from these two clusters, as well as from clusters 3 and 4, were significantly higher expressed in the group of natalizumab-treated patients, whereas they were lower expressed in the cladribine group and, at least in the case of cluster 2 and cluster 4 genes, also the alemtuzumab group. Conversely, the clusters 7 and 8 contained genes that were expressed at low levels in the natalizumab group but at relatively high levels in the alemtuzumab group and the cladribine group. The expression profiles of the healthy subjects, the PPMS patients and the before IRT group were fairly similar. Only a few genes were generally decreased (*n* = 5, cluster 5) or increased (*n* = 7, cluster 6) in expression in the enriched B cells of the patients with MS as compared to those of the healthy controls.

For a closer look, we investigated the expression changes in RRMS patients treated with alemtuzumab by analyzing the data of the 21 paired samples separately. By this means, we identified 225, 86 and 44 genes with significantly altered expression in response to the first, second and third treatment course, respectively (Additional file 1: Fig. S3, Additional file 4). Of the top 500 DEGs, a subset of 105 genes were significantly downregulated (*n* = 79) or upregulated (*n* = 26) from B1 to F1 according to the filtering criteria (*t* test *p* < 0.05 and log2FC <  − 1 or > 1).

### Gene functions and cell type specificity

To gain insights into the modulated biological processes, we tested for each of the 8 clusters whether the DEGs were significantly overrepresented in GO categories. The most significant enrichment was seen for the GO term "RNA processing". A total of 74 genes from cluster 1 belonged to this category, e.g., *BCAS2*, *RMRP*, *RPPH1* as well as genes encoding ribosomal proteins and small nuclear/nucleolar RNAs (Fig. 4 and Additional file 5). Cluster 2 genes were significantly related to "transport" (e.g., *ADRB2*, *ANKH* and *IL10RA*) and "positive regulation of metabolic process" (e.g., *CALM2*, *PARP3* and *RIPK2*). Other GO terms that were associated with the top DEGs were "apoptotic process" (cluster 3, e.g., *BCL2*, *IGF1R* and *NFKBIA*), "regulation of signal transduction" (cluster 4, e.g., *CD80*, *FCGR2B* and *RGS7*), "cell adhesion" (cluster 6, *PCDH9*, *PDLIM1* and *PTPRK*) and "immune system process" (cluster 8, e.g., *CD1A*, *CR2* and *IL21R*).

Expression data for B-cell subsets were available for 454 of the top 500 DEGs in the data set from Monaco et al. [54] (Additional file 4). We used these information to examine the cell type specificity for each cluster. As can be seen in Fig. 4, the genes from clusters 2 and 4, which were reduced in expression in RRMS patients treated with alemtuzumab or cladribine, are predominantly expressed by memory B cells. On the other hand, the genes from cluster 8, which were increased in expression in those patients who were treated with an IRT, are primarily expressed by naive B cells. Cluster 7, which contains genes that were expressed at much lower levels in patients receiving natalizumab infusions, showed an association to the expression signature of plasmablasts. These data agree well with the differences in the composition of circulating B-cell subpopulations between the study groups that we have measured by flow cytometry.

### Interaction network of differentially expressed genes

**Fig. 5:**
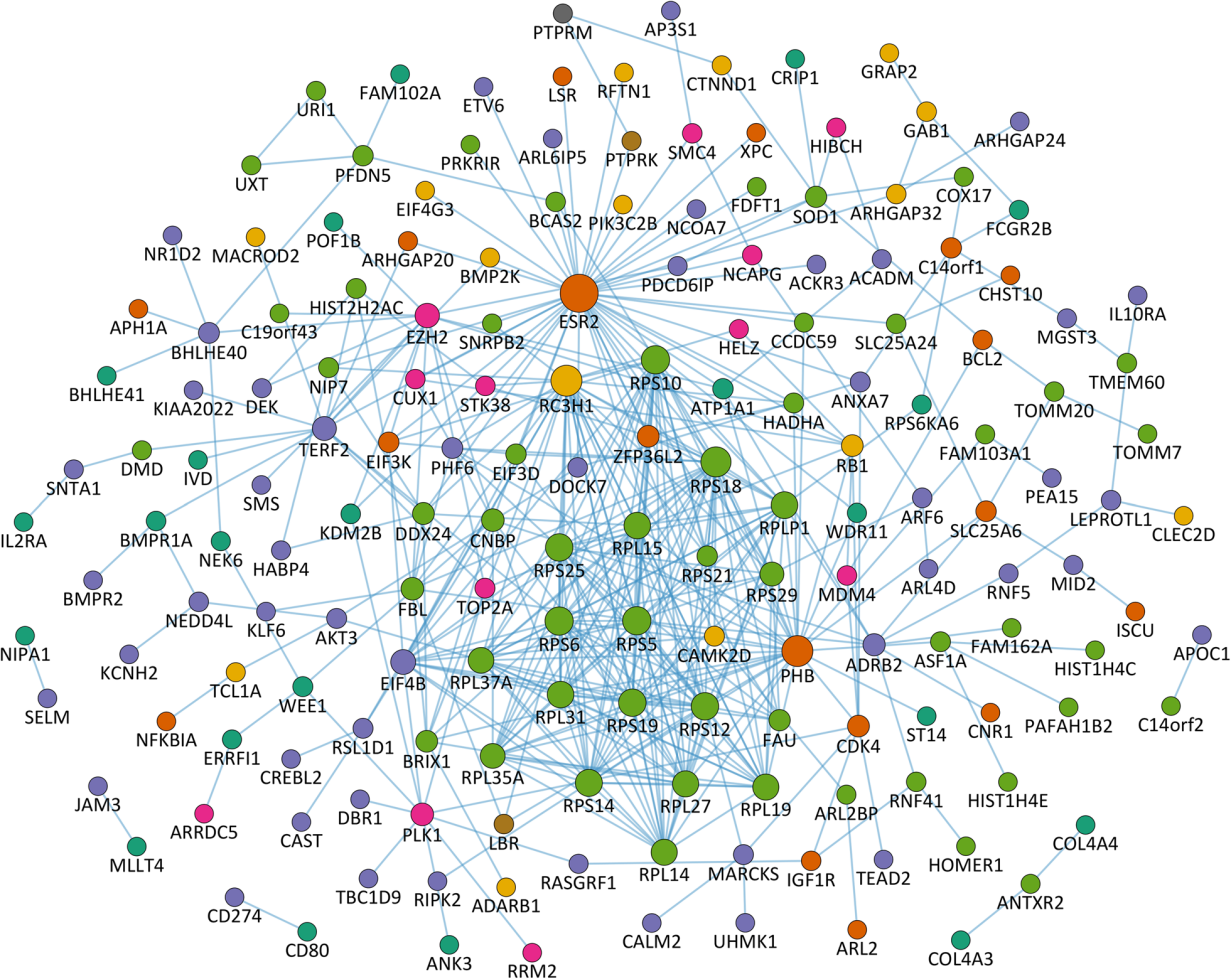
Protein–protein interaction network of differentially expressed genes. The gene symbols of the top 500 differentially expressed genes (DEGs) were entered in the GeneMANIA Cytoscape plugin [57, 58]. The search identified 428 physical interactions (blue edges) between 173 gene products (nodes colored by cluster membership). The other top DEGs were not linked by protein–protein interactions and are thus not shown in the network. The size of the nodes corresponds to the number of edges

We next explored protein–protein interactions between the gene products of the top 500 DEGs using the GeneMANIA plugin for Cytoscape [57, 58]. The analysis yielded a total of 428 physical interactions between 173 of the top DEGs (Fig. 5). The most connected proteins in the network were ESR2 (45 edges), PHB (29 edges) and RC3H1 (29 edges). Between small and large ribosomal subunit proteins (*n* = 10 and *n* = 8, respectively) from cluster 1, there was a particularly high number of pairwise interactions (129 edges in the network).

### Relapse-associated gene expression variation

Finally, we searched for genes whose transcript levels in B cells might have a predictive value with regard to disease activity despite IRT with alemtuzumab. Two of the 4 patients for whom samples were available before and after the first treatment course had a relapse in the year after the B1 timepoint, 4 of the 13 patients with B2–F2 sample pairs experienced a relapse in the year after the administration of the second treatment course, and all three patients with B3–F3 sample pairs had a relapse in the year following B3 (Additional file 2). The average time to relapse was 8.4 ± 3.0 months. Age (*t* test *p* = 0.597), sex (Fisher's exact test *p* = 1.000), disease duration (*U* test *p* = 0.776), EDSS score (*t* test *p* = 0.689) and the number of relapses in the preceding year (*t* test *p* = 0.457) were not significantly associated with the occurrence of clinical relapses in the follow-up year.

**Fig. 6:**
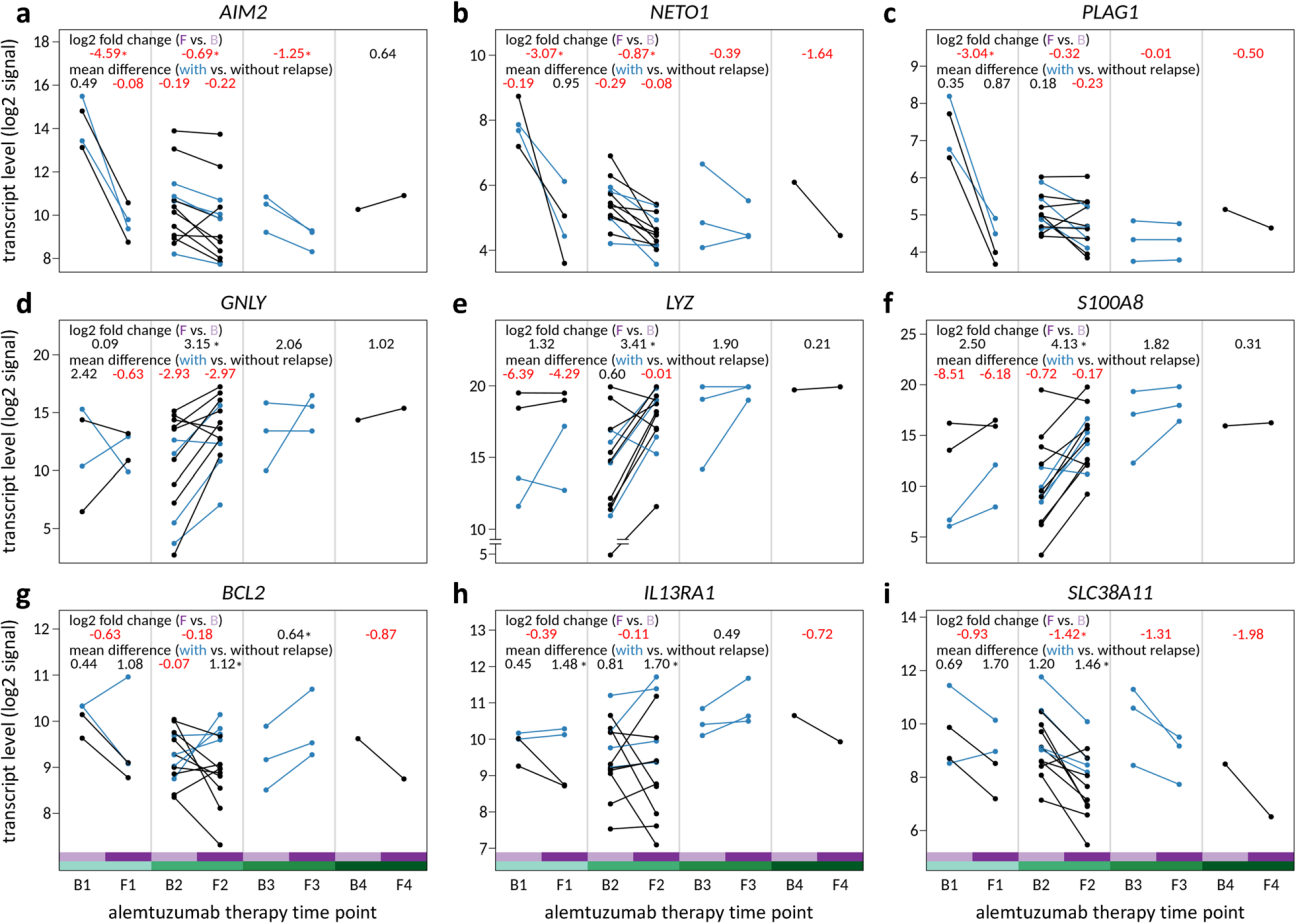
Expression dynamics during alemtuzumab therapy for selected genes. Shown are mRNA levels in B cells immediately before (B) as well as ~ 7 months following (F) the 1st, 2nd, 3rd or 4th alemtuzumab treatment course. Lines connect the data for the B sample and the F sample from the same patient (*n* = 21 sample pairs). Blue dots/lines indicate that the patient experienced a relapse in the 12 months after drug administration (*n* = 2 for the first treatment course and *n* = 4 for the second treatment course), while black dots/lines indicate that the patient was free of relapses in the follow-up period. **a**–**c** Genes expressed at an extremely lower level (log2 fold change <  − 3 and *p* < 0.05) following the start of alemtuzumab therapy. **d–f** Genes expressed at an extremely higher level (log2 fold change > 3 and *p* < 0.05) after the second treatment course. **g**–**i** Genes differentially expressed between patients with and without relapse (mean difference > 1 at F1 and F2 and *p* < 0.05 at one timepoint). Negative values are displayed in red. The data for further interesting genes are shown in Additional file 1: Fig. S4. * p < 0.05

We first evaluated whether genes with extreme shifts in expression after alemtuzumab infusions were differentially expressed when comparing patients with and without relapse. There were 5 genes (*AIM2*, *BHLHE41*, *NETO1*, *PLAG1* and *TFEC*) that were expressed at extremely lower levels after the first treatment course (*t* test *p* < 0.05 and log2FC <  − 3) and 5 genes (*CX3CR1*, *GNLY*, *LYZ*, *S100A8* and *S100A9*) that were expressed at extremely higher levels after the second treatment course (*t* test *p* < 0.05 and log2FC > 3). The gene expression dynamics are shown in Fig. 6a–f and Additional file 1: Fig. S4a–d. However, the mRNA levels of these 10 genes were not found to be associated with relapse risk. Despite MD of up to 8.51 in the log2 signal intensities, the significance level could not be reached at any timepoint (*t* test *p* > 0.05) (Additional file 4). There was also no significant difference in the expression changes of these genes from B1 to F1 and from B2 to F2 between patients with relapse and patients without relapse.

After we extended the analysis to all transcript clusters, we found a differential expression of 242 genes (*p* < 0.05 and MD <  − 1 or > 1 at B1, F1, B2 or F2), with the more stringent selection criteria being met by 17 of these genes, but none remained after FDR correction. Six of the 17 genes (*DMXL2*, *GSN*, *MIR4435-2HG*, *RARRES3*, *RNU12* and *TIMP1*) had a lower expression in the B cells from the patients with relapse event in the year following an alemtuzumab treatment course. The other 11 genes were expressed at higher levels at two timepoints (MD > 1), while reaching the significance level at one timepoint (*p* < 0.05), when comparing alemtuzumab-treated patients with and without a relapse. Figure 6g-–i shows the data for 3 of the 11 genes (*BCL2*, *IL13RA1* and *SLC38A11*). The data for the other filtered genes are shown in Additional file 1: Fig. S4e–r. The mean differences and *p *values are provided for all genes in Additional file 4.

## Discussion

Over the past years, evidence has accumulated that B cells and their interplay with T cells are central in the pathogenesis of MS [25]. Our understanding of this complex disease has considerably advanced with the success of therapies that mediate the depletion or functional inhibition of immune cells [29]. Previous studies have characterized the shifts at the cellular level that occur during the treatment with pulsed IRTs and anti-CD20 agents [59]. Both alemtuzumab and cladribine induce a rapid depletion of lymphocytes, after which memory B cells repopulate only slowly and thus are persistently depleted in the blood of patients with MS [30, 31, 33–36]. This is thought to reduce B-cell trafficking from the periphery to the CNS, antigen presentation to T cells, pro-inflammatory cytokine production and the generation of antibody-secreting cells [8]. Here, we utilized a transcriptomics approach to obtain more detailed insights on the therapeutic effects at the molecular level. We explored the biological processes that are influenced as a consequence of the gene expression alterations following the administration of IRTs and filtered biomarker candidates of the effectiveness of alemtuzumab treatment in preventing relapses.

Our analysis was based on 121 blood samples that were collected from 91 subjects and divided into 6 study groups. We included a healthy group, a PPMS group and four RRMS subgroups (before IRT, alemtuzumab, cladribine and natalizumab). The patient cohort was typical for this disease in terms of age, sex and degree of disability [42], but we did not include patients with SPMS, patients who were therapy-naive and patients treated with other DMTs, such as ocrelizumab. Moreover, older individuals were underrepresented in the healthy group, which may have impacted the results. However, age-related changes in the proportions of B-cell subsets are most pronounced in the first 5 years of life [46], whereas in adults, two reference studies generally found no statistically significant change with age, even though a marked decrease in CD27^+^IgD^+^ B cells and plasmablasts was observed [60, 61]. Of note, the patients who received alemtuzumab would have met the inclusion criteria of the respective phase III clinical trials in terms of age, EDSS score, course of MS and number of relapses in the pre-treatment phase. The patients from whom we obtained a B1 sample before starting cladribine therapy, however, would not have met the criterion of having at least one relapse in the previous 12 months. This resembles the finding that the number of relapses before treatment is the most frequent clinical trial criterion that is not fulfilled in routine clinical care [62]. The therapies with alemtuzumab and cladribine are referred to as pulsed IRTs as they induce a partial immune reset to achieve long-term drug-free remission of disease activity, which is a different concept compared to therapies that need to be given continuously to maintain their therapeutic efficacy [10]. However, despite the high efficacy of IRTs in the relative reduction in relapse risk [63], relapses still occur in some patients following treatment with alemtuzumab [64] or cladribine [65]. Some patients thus require retreatment with alemtuzumab. In our collection, we had samples from 4 patients who received a 3^rd^ or 4^th^ course of alemtuzumab, because they had 1 or 2 relapses in the past year. For practical reasons, we included patients before and after different treatment courses of alemtuzumab and cladribine. A longitudinal blood collection for each patient from the beginning of IRT and across multiple timepoints in the subsequent years would have been more appropriate but difficult to implement.

Our study focused on B cells as they are major contributors to the immune responses involved in MS [25]. In addition to the transcriptome profiling, we used flow cytometry to characterize the B cells from the peripheral blood. When we compared the healthy controls with the RRMS patients before IRT, we could find no difference in the frequencies of the distinct B-cell subsets (Tukey test *p* > 0.05). However, substantial B-cell subpopulation shifts were apparent in the treatment groups. The patients who received a pulsed IRT showed significantly higher proportions of transitional and naive B cells and much lower proportions of memory B cells, which is consistent with earlier studies on the effects of alemtuzumab [30, 31] and cladribine [33–36]. In contrast, the therapy with natalizumab, an antibody to α4 integrins, leads to a preferential expansion of the memory B-cell pool, which is attributable to a decreased retention of these cells within secondary lymphoid tissues [66–68]. At the same time, the proportion of CD21^−/low^CD38^−/low^ B cells, which have been shown to be enriched with autoreactive unresponsive clones in some autoimmune diseases [47, 69], was significantly lower in patients on IRTs but significantly higher in patients on natalizumab therapy. Further research on CNS-resident and antigen-specific B cells may provide deeper insights into the therapeutic mechanisms of action. Besides, IRTs for MS also have effects on T cells and to a lesser extent on circulating cells of the innate immune system [9, 30–34, 70], which also deserve to be explored in more detail at the cellular and transcriptome level.

To our knowledge, the B-cell transcriptomes of MS patients undergoing IRTs were measured for the first time in our study. This was done using Clariom D arrays, which were introduced in 2016 as successor of previous high-density microarray solutions [71]. These arrays are highly reproducible in estimating gene and exon levels, and they allow to detect even small variations in expression, especially for low-abundant transcripts [72]. However, as a limitation, they offer a lower dynamic range than RNA sequencing and cannot provide insights into the expression of single cells. In comparison of the 6 study groups, a total of 6,280 DEGs resulted after FDR correction, and we took a closer look at the top 500 DEGs. Remarkably, except for the few genes in cluster 5 and cluster 6, the expression profiles were relatively similar between healthy subjects, PPMS patients (who were treated) and RRMS patients before IRT (who just discontinued another DMT). However, strong and opposite transcriptome alterations were observed for the IRT groups and the natalizumab group. These gene expression differences are essentially a consequence of the treatment-related shifts in B-cell subsets. Following IRT (i.e., after B-cell depletion and B-cell repopulation), the majority of the DEGs were reduced in expression, while cluster 8 genes were expressed at much higher levels and clearly related to the expression signature of naive B cells. Differences between the alemtuzumab group and the cladribine group were rather confined to the expression of cluster 1 and cluster 3 genes. The analysis of the paired samples revealed that the transcriptome changes in response to alemtuzumab primarily occurred after the first treatment course, whereas there were smaller effects on gene expression after the second and third annual course. We suspect that the response to cladribine is also strongest after the first course, but we could not verify this because of the variable timing of blood withdrawals and the small number of samples in this group.

Our data show that the B-cell composition that reconstitutes following IRT is functionally different from that before IRT and from that of MS patients on other therapies. Among the top 500 DEGs, there were several genes that are involved in the activation of lymphocytes. For instance, *CR2* (from cluster 8), which encodes CD21, a cell surface receptor for complement C3 and for EBV on human B cells [73], was significantly higher expressed in patients treated with alemtuzumab or cladribine. In these patients, we also measured lower mRNA levels of *FCGR2B* (cluster 4), which encodes a receptor for the Fc region of immunoglobulin gamma complexes that inhibits B-cell receptor (BCR) signaling and antibody production [74]. Moreover, in those patients who received an IRT, we observed an increased expression of *CD1A* (cluster 8) and a reduced expression of *CD80* (cluster 4), which encode membrane proteins that play a role in T-cell activation by B cells and other immune cells [75, 76]. Transcripts for the cytokine receptors IL10RA (cluster 2) and IL21R (cluster 8) were also found to be differentially expressed between the study groups. IL10RA, which appeared to be expressed at lower levels in B cells of MS patients compared to healthy controls as previously reported [77], mediates the immunosuppressive signal of IL10 by inhibiting the expression of pro-inflammatory genes [78]. IL21R, which is predominantly expressed by naive B cells and was thus increased in expression in patients treated with an IRT, transduces the signal of IL21, which is produced by T-cell subsets and regulates the proliferation and differentiation of B cells and antibody responses [79]. Serum levels of IL21 have been proposed as biomarker for the risk of developing secondary autoimmunity following alemtuzumab treatment [80]. However, we could not study this issue on the basis of our data. Other DEGs were found to regulate apoptotic processes. For instance, CALM2 (from cluster 2) is an intracellular calcium-binding protein involved in cell death upon BCR stimulation [81], RIPK2 (cluster 2) is a serine/threonine protein kinase suppressing apoptosis by regulating nuclear factor κB signaling [82], and IGF1R (cluster 3) is a receptor with tyrosine kinase activity that is known to mediate anti-apoptotic effects via the PI3K/AKT pathway [83]. We observed the lowest average mRNA expression of these genes in the cladribine group (*CALM2* and *RIPK2*) and the alemtuzumab group (*IGF1R*), respectively. Cluster 1, in turn, was significantly associated with the GO term "RNA processing", because it contains protein-coding and non-coding genes that promote the splicing of pre-mRNAs (e.g., *BCAS2* and *RNU6-1*) [84] and the biogenesis of transfer RNAs and ribosomal RNAs (e.g., *RMRP*, *RPPH1* and small nucleolar RNAs) [85–87]. In addition, genes encoding ribosomal proteins belong to this cluster. In the MS patient subgroups, we also detected an increased expression of genes regulating cell contact and adhesion (e.g., *PCDH9*, *PDLIM1* and *PTPRK* from cluster 6) [88–90]. Among the most highly connected genes in the interaction network were *ESR2* and *PHB* (cluster 3, low in cladribine group) and *RC3H1* (cluster 8, low in natalizumab group). The estrogen receptor ESR2 and the ubiquitously expressed protein PHB are regulators of transcription [91, 92]. Furthermore, PHB is involved in CD86 signaling in B cells [93] and in the correct folding of mitochondrial proteins [94]. RC3H1 is a post-transcriptional repressor of mRNAs (e.g., *IL6* mRNA) [95] and also regulates the decay of microRNAs (e.g., miR-146a) [96]. Of note, we have focused here on the top 500 DEGs, even though the expression shifts under IRT were much broader. In a recent study, Moser et al. reported reduced proportions of CD19^+^ B cells with CD44, ITGA4, ITGAL, ITGB1 and HLA-DR surface expression at 24 months after the initiation of cladribine therapy [97]. In our analysis, those genes were not among the top 500 DEGs, but they were differentially expressed with FDR < 0.05 and all of them had the lowest average expression in the cladribine group. Thus, our data confirm their results from flow cytometry measurements at the transcript level.

We used the B-cell transcriptome profiles to search biomarkers for identifying patients with active disease following the administration of alemtuzumab. Although no gene remained significant after adjustment for multiple testing, our stringent selection resulted in 17 genes whose expression differed substantially when comparing patients with relapse and patients without relapse in the year after the 1st or 2nd alemtuzumab treatment course. This analysis was limited by the small number of patients per group. Nevertheless, we consider the genes to be reasonable candidates for further confirmatory studies at the RNA or protein level. For instance, in relapse-free patients, *BCL2* was in most cases decreased in expression at the follow-up timepoints, whereas in patients with relapse, its expression was usually increased, which resulted in a more than twofold higher average expression in these patients at F1 and F2, respectively. *BCL2* encodes a key anti-apoptotic protein that controls mitochondrial outer membrane permeability [98]. The apoptosis pathway that is regulated by the Bcl-2 protein family is critical for lymphocyte development, maintenance of peripheral tolerance and prevention of autoimmunity [99], and Bcl-2 family antagonism has been demonstrated to be a potential approach for the treatment of autoimmune diseases [100]. It is thus possible that lower mRNA levels of *BCL2* in response to IRT may correlate with reduced disease activity in patients with MS. Another interesting gene is *IL13RA1*, which was also expressed at higher levels in patients who relapsed. *IL13RA1* encodes a receptor subunit that mediates the signaling events induced by IL13 [101]. Previous studies reported significantly higher percentages of IL13-producing T cells in the blood and CSF of patients in relapse compared to patients in remission [102, 103]. Similarly, higher levels of the receptor might, therefore, be related to a higher risk of clinical relapse due to a suboptimal disease control. We also observed higher levels of *SLC38A11* in alemtuzumab-treated patients experiencing a relapse, while the average expression decreased after each treatment course. SLC38A11 is a member of the SLC38 family of transmembrane sodium-coupled amino acid transporters, which are particularly expressed in cells that carry out significant amino acid metabolism [104, 105]. However, its role in B cells and MS is still unclear. In the interpretation of our results, it should be noted that early disease activity after initiation of IRT does not necessarily implicate treatment failure and that it is usually appropriate to continue the therapy. For example, one of our patients had 3 relapses in the year before IRT and another relapse in the first year of alemtuzumab therapy but was relapse-free in the second year. This patient received a pre-treatment with fingolimod, which has been reported to be a risk factor of relapses following alemtuzumab infusion [106]. Further research is needed to study the relationship of gene expression signatures in the blood and specific treatment sequences with the individual course of disease. This should help to translate potential candidates into clinically useful molecular biomarkers and to guide more personalized therapeutic decisions in the near future.

A hallmark but also a limitation of the present study is the sole focus on B cells. Furthermore, the source of RNA for the transcriptome analysis was a mixture of B-cell subsets. Meanwhile, the recent rise of single-cell multi-omics technologies has enabled researchers not only to study gene expression patterns at the single-cell level but also to obtain information on the (epi)genetics and proteomics of individual cells at the same time [107]. Others used RNA sequencing to investigate the temporal dynamics in B-cell immunoglobulin heavy chain repertoires during IRT [108, 109]. Through integration of such different types of data, together with metabolomic profiles, it should be possible to better define perturbations in the immune signature of patients with MS. Further advances in our understanding of the disease processes will ultimately drive the development of even more selective, effective and safe therapeutics for MS. This may bring us closer to the goal of preventing neurological deterioration and inducing long-lasting drug-free disease stability. Another limitation of our study is the rather small number of patients per therapy timepoint. Therefore, the identification of potential gene expression markers of relapse activity in alemtuzumab-treated patients was exploratory in nature. Moreover, we did not analyze other treatment outcomes, such as MRI findings and the development of secondary autoimmune disorders, because the available data were too sparse and heterogeneous. Additional studies are required to confirm that therapeutic efficacy correlates with the expression of genes that we have nominated as biomarker candidates. If they prove to be useful for prognosis and monitoring of disease activity, they may allow to select patients who will benefit most from an IRT and/or patients who need an additional treatment course.

## Conclusions

We demonstrate that the B-cell transcriptome is substantially reorganized already after the first course of an IRT. Similar effects were seen under therapy with alemtuzumab and cladribine, although some genes were reduced in expression most markedly in the cladribine group (cluster 1 and cluster 3). Opposite gene expression alterations were found for RRMS patients who received natalizumab. These expression patterns are largely explained by the therapy-induced shifts in the proportions of naive and memory B-cell subpopulations and implicate a functionally different adaptive immune profile. More specifically, the top 500 DEGs were found to participate in, for example, lymphocyte activation, apoptotic signaling, RNA processing and cellular adhesion. We could also relate the occurrence of relapses following alemtuzumab infusions with the transcript levels of 17 genes, which qualifies them as potential indicators of the clinical response to therapy. Our study may inform further research toward gaining deeper insights into MS-associated immune mechanisms and developing improved treatment approaches that are tailored to the pathobiologic phenotype of individual patients.

## Supplementary Information

**Additional file 1.** Gating strategy for the B-cell phenotyping as well as cell population shifts and expression dynamics during alemtuzumab therapy.

**Additional file 2.** Information about the 121 blood samples with clinical-demographic data of the patients and assignments to the GEO data set GSE190847.

**Additional file 3.** Comparison of CD19^+^ B-cell subpopulation frequencies between the 6 study groups.

**Additional file 4.** The transcriptome data for B cells of patients with MS and healthy controls with average expression levels per group, results of the differential gene expression analyses and cluster memberships.

**Additional file 5.** Enrichment of Gene Ontology terms for each gene cluster.

## Data Availability

The data supporting the findings of this study are available in the article and/or supplementary materials. The B-cell transcriptome data are publicly available from the GEO database (https://www.ncbi.nlm.nih.gov/geo/query/acc.cgi?acc=GSE190847).

## Abbreviations

APC: Allophycocyanin
B: Before
B ex: Exhausted memory B cells
B n: Naive B cells
B nsm: Non-switched memory B cells
B sm: Switched memory B cells
BCR: B-cell receptor
BV: Brilliant violet
CNS: Central nervous system
CSF: Cerebrospinal fluid
DEG: Differentially expressed gene
DMT: Disease-modifying therapy
EBV: Epstein–Barr virus
EDSS: Expanded Disability Status Scale
EDTA: Ethylenediaminetetraacetic acid
F: Following
FC: Fold change
FDR: False discovery rate
GEO: Gene Expression Omnibus
GO: Gene Ontology
IRT: Immune reconstitution therapy
LMM: Linear mixed-effects model
MD: Mean difference
MRI: Magnetic resonance imaging
mRNA: Messenger RNA
MS: Multiple sclerosis
pb: Plasmablasts
PBMC: Peripheral blood mononuclear cells
PE: Phycoerythrin
PerCP: Peridinin–chlorophyll
PPMS: Primary progressive multiple sclerosis
RRMS: Relapsing–remitting multiple sclerosis
SD: Standard deviation
SPMS: Secondary progressive multiple sclerosis

## References

[1] Filippi M, Bar-Or A, Piehl F, Preziosa P, Solari A, Vukusic S, Rocca MA (2018). Multiple sclerosis. Nat Rev Dis Primers.

[2] Walton C, King R, Rechtman L, Kaye W, Leray E, Marrie RA, Robertson N, La Rocca N, Uitdehaag B, van der Mei I, Wallin M, Helme A, Angood Napier C, Rijke N, Baneke P (2020). Rising prevalence of multiple sclerosis worldwide: insights from the Atlas of MS, third edition. Mult Scler.

[3] Thompson AJ, Banwell BL, Barkhof F, Carroll WM, Coetzee T, Comi G, Correale J, Fazekas F, Filippi M, Freedman MS, Fujihara K, Galetta SL, Hartung HP, Kappos L, Lublin FD, Marrie RA, Miller AE, Miller DH, Montalban X, Mowry EM, Sorensen PS, Tintoré M, Traboulsee AL, Trojano M, Uitdehaag BMJ, Vukusic S, Waubant E, Weinshenker BG, Reingold SC, Cohen JA (2018). Diagnosis of multiple sclerosis: 2017 revisions of the McDonald criteria. Lancet Neurol.

[4] Lublin FD, Reingold SC, Cohen JA, Cutter GR, Sørensen PS, Thompson AJ, Wolinsky JS, Balcer LJ, Banwell B, Barkhof F, Bebo B, Calabresi PA, Clanet M, Comi G, Fox RJ, Freedman MS, Goodman AD, Inglese M, Kappos L, Kieseier BC, Lincoln JA, Lubetzki C, Miller AE, Montalban X, O'Connor PW, Petkau J, Pozzilli C, Rudick RA, Sormani MP, Stüve O, Waubant E, Polman CH (2014). Defining the clinical course of multiple sclerosis: the 2013 revisions. Neurology.

[5] Olsson T, Barcellos LF, Alfredsson L (2017). Interactions between genetic, lifestyle and environmental risk factors for multiple sclerosis. Nat Rev Neurol.

[6] Hecker M, Bühring J, Fitzner B, Rommer PS, Zettl UK (2021). Genetic, environmental and lifestyle determinants of accelerated telomere attrition as contributors to risk and severity of multiple sclerosis. Biomolecules.

[7] McGinley MP, Goldschmidt CH, Rae-Grant AD (2021). Diagnosis and treatment of multiple sclerosis: a review. JAMA.

[8] Hauser SL, Cree BAC (2020). Treatment of multiple sclerosis: a review. Am J Med.

[9] Rommer PS, Milo R, Han MH, Satyanarayan S, Sellner J, Hauer L, Illes Z, Warnke C, Laurent S, Weber MS, Zhang Y, Stuve O (2019). Immunological aspects of approved MS therapeutics. Front Immunol.

[10] Sorensen PS, Sellebjerg F (2019). Pulsed immune reconstitution therapy in multiple sclerosis. Ther Adv Neurol Disord.

[11] Lünemann JD, Ruck T, Muraro PA, Bar-Or A, Wiendl H (2020). Immune reconstitution therapies: concepts for durable remission in multiple sclerosis. Nat Rev Neurol.

[12] Coles AJ, Twyman CL, Arnold DL, Cohen JA, Confavreux C, Fox EJ, Hartung HP, Havrdova E, Selmaj KW, Weiner HL, Miller T, Fisher E, Sandbrink R, Lake SL, Margolin DH, Oyuela P, Panzara MA, Compston DA, CARE-MS II investigators (2012). Alemtuzumab for patients with relapsing multiple sclerosis after disease-modifying therapy: a randomised controlled phase 3 trial. Lancet.

[13] Berger T, Elovaara I, Fredrikson S, McGuigan C, Moiola L, Myhr KM, Oreja-Guevara C, Stoliarov I, Zettl UK (2017). Alemtuzumab use in clinical practice: recommendations from european multiple sclerosis experts. CNS Drugs.

[14] Ruck T, Bittner S, Wiendl H, Meuth SG (2015). Alemtuzumab in multiple sclerosis: mechanism of action and beyond. Int J Mol Sci.

[15] Giovannoni G, Comi G, Cook S, Rammohan K, Rieckmann P, SoelbergSørensen P, Vermersch P, Chang P, Hamlett A, Musch B, Greenberg SJ, CLARITY Study Group (2010). A placebo-controlled trial of oral cladribine for relapsing multiple sclerosis. N Engl J Med.

[16] Wiendl H (2017). Cladribine—an old newcomer for pulsed immune reconstitution in MS. Nat Rev Neurol.

[17] Montalban X, Hauser SL, Kappos L, Arnold DL, Bar-Or A, Comi G, de Seze J, Giovannoni G, Hartung HP, Hemmer B, Lublin F, Rammohan KW, Selmaj K, Traboulsee A, Sauter A, Masterman D, Fontoura P, Belachew S, Garren H, Mairon N, Chin P, Wolinsky JS, ORATORIO Clinical Investigators (2017). Ocrelizumab versus placebo in primary progressive multiple sclerosis. N Engl J Med.

[18] Lamb YN (2022). Ocrelizumab: a review in multiple sclerosis. Drugs.

[19] Winkelmann A, Loebermann M, Reisinger EC, Hartung HP, Zettl UK (2016). Disease-modifying therapies and infectious risks in multiple sclerosis. Nat Rev Neurol.

[20] Wiendl H, Gold R, Berger T, Derfuss T, Linker R, Mäurer M, Aktas O, Baum K, Berghoff M, Bittner S, Chan A, Czaplinski A, Deisenhammer F, Di Pauli F, Du Pasquier R, Enzinger C, Fertl E, Gass A, Gehring K, Gobbi C, Goebels N, Guger M, Haghikia A, Hartung HP, Heidenreich F, Hoffmann O, Kallmann B, Kleinschnitz C, Klotz L, Leussink VI, Leutmezer F, Limmroth V, Lünemann JD, Lutterotti A, Meuth SG, Meyding-Lamadé U, Platten M, Rieckmann P, Schmidt S, Tumani H (2021). Multiple sclerosis therapy consensus group (MSTCG): position statement on disease-modifying therapies for multiple sclerosis (white paper). Ther Adv Neurol Disord.

[21] Soldan SS, Lieberman PM (2023). Epstein–Barr virus and multiple sclerosis. Nat Rev Microbiol.

[22] Bjornevik K, Cortese M, Healy BC, Kuhle J, Mina MJ, Leng Y, Elledge SJ, Niebuhr DW, Scher AI, Munger KL, Ascherio A (2022). Longitudinal analysis reveals high prevalence of Epstein–Barr virus associated with multiple sclerosis. Science.

[23] Abrahamyan S, Eberspächer B, Hoshi MM, Aly L, Luessi F, Groppa S, Klotz L, Meuth SG, Schroeder C, Grüter T, Tackenberg B, Paul F, Then-Bergh F, Kümpfel T, Weber F, Stangel M, Bayas A, Wildemann B, Heesen C, Zettl U, Warnke C, Antony G, Hessler N, Wiendl H, Bittner S, Hemmer B, Gold R, Salmen A, Ruprecht K, German Competence Network Multiple Sclerosis (KKNMS); Other members of the KKNMS that acted as collaborators in this study (2020). Complete Epstein–Barr virus seropositivity in a large cohort of patients with early multiple sclerosis. J Neurol Neurosurg Psychiatry.

[24] Cencioni MT, Mattoscio M, Magliozzi R, Bar-Or A, Muraro PA (2021). B cells in multiple sclerosis—from targeted depletion to immune reconstitution therapies. Nat Rev Neurol.

[25] Comi G, Bar-Or A, Lassmann H, Uccelli A, Hartung HP, Montalban X, Sørensen PS, Hohlfeld R, Hauser SL, Expert Panel of the 27th Annual Meeting of the European Charcot Foundation (2021). Role of B cells in multiple sclerosis and related disorders. Ann Neurol.

[26] Jelcic I, Al Nimer F, Wang J, Lentsch V, Planas R, Jelcic I, Madjovski A, Ruhrmann S, Faigle W, Frauenknecht K, Pinilla C, Santos R, Hammer C, Ortiz Y, Opitz L, Grönlund H, Rogler G, Boyman O, Reynolds R, Lutterotti A, Khademi M, Olsson T, Piehl F, Sospedra M, Martin R (2018). Memory B cells activate brain-homing, autoreactive CD4^+^ T cells in multiple sclerosis. Cell.

[27] Fraussen J, Claes N, Van Wijmeersch B, van Horssen J, Stinissen P, Hupperts R, Somers V (2016). B cells of multiple sclerosis patients induce autoreactive proinflammatory T cell responses. Clin Immunol.

[28] Guo MH, Sama P, LaBarre BA, Lokhande H, Balibalos J, Chu C, Du X, Kheradpour P, Kim CC, Oniskey T, Snyder T, Soghoian DZ, Weiner HL, Chitnis T, Patsopoulos NA (2022). Dissection of multiple sclerosis genetics identifies B and CD4+ T cells as driver cell subsets. Genome Biol.

[29] Baker D, Marta M, Pryce G, Giovannoni G, Schmierer K (2017). Memory B cells are major targets for effective immunotherapy in relapsing multiple sclerosis. EBioMedicine.

[30] Thompson SA, Jones JL, Cox AL, Compston DA, Coles AJ (2010). B-cell reconstitution and BAFF after alemtuzumab (Campath-1H) treatment of multiple sclerosis. J Clin Immunol.

[31] Baker D, Herrod SS, Alvarez-Gonzalez C, Giovannoni G, Schmierer K (2017). Interpreting lymphocyte reconstitution data from the pivotal Phase 3 trials of alemtuzumab. JAMA Neurol.

[32] Rolfes L, Pfeuffer S, Huntemann N, Schmidt M, Su C, Skuljec J, Aslan D, Hackert J, Kleinschnitz K, Hagenacker T, Pawlitzki M, Ruck T, Kleinschnitz C, Meuth SG, Pul R (2022). Immunological consequences of cladribine treatment in multiple sclerosis: a real-world study. Mult Scler Relat Disord..

[33] Moser T, Schwenker K, Seiberl M, Feige J, Akgün K, Haschke-Becher E, Ziemssen T, Sellner J (2020). Long-term peripheral immune cell profiling reveals further targets of oral cladribine in MS. Ann Clin Transl Neurol.

[34] Baker D, Pryce G, Herrod SS, Schmierer K (2019). Potential mechanisms of action related to the efficacy and safety of cladribine. Mult Scler Relat Disord.

[35] Wiendl H, Schmierer K, Hodgkinson S, Derfuss T, Chan A, Sellebjerg F, Achiron A, Montalban X, Prat A, De Stefano N, Barkhof F, Leocani L, Vermersch P, Chudecka A, Mwape C, Holmberg KH, Boschert U, Roy S (2023). Specific patterns of immune cell dynamics may explain the early onset and prolonged efficacy of cladribine tablets: a MAGNIFY-MS substudy. Neurol Neuroimmunol Neuroinflamm..

[36] Ceronie B, Jacobs BM, Baker D, Dubuisson N, Mao Z, Ammoscato F, Lock H, Longhurst HJ, Giovannoni G, Schmierer K (2018). Cladribine treatment of multiple sclerosis is associated with depletion of memory B cells. J Neurol.

[37] Duddy M, Niino M, Adatia F, Hebert S, Freedman M, Atkins H, Kim HJ, Bar-Or A (2007). Distinct effector cytokine profiles of memory and naive human B cell subsets and implication in multiple sclerosis. J Immunol.

[38] Baker D, Herrod SS, Alvarez-Gonzalez C, Zalewski L, Albor C, Schmierer K (2017). Both cladribine and alemtuzumab may effect MS via B-cell depletion. Neurol Neuroimmunol Neuroinflamm..

[39] Kousin-Ezewu O, Azzopardi L, Parker RA, Tuohy O, Compston A, Coles A, Jones J (2014). Accelerated lymphocyte recovery after alemtuzumab does not predict multiple sclerosis activity. Neurology.

[40] Gilmore W, Lund BT, Li P, Levy AM, Kelland EE, Akbari O, Groshen S, Cen SY, Pelletier D, Weiner LP, Javed A, Dunn JE, Traboulsee AL (2020). Repopulation of T, B, and NK cells following alemtuzumab treatment in relapsing-remitting multiple sclerosis. J Neuroinflamm.

[41] Wiendl H, Carraro M, Comi G, Izquierdo G, Kim HJ, Sharrack B, Tornatore C, Daizadeh N, Chung L, Jacobs AK, Hogan RJ, Wychowski LV, Van Wijmeersch B, CARE-MS I, CARE-MS II, and CAMMS03409 Investigators (2019). Lymphocyte pharmacodynamics are not associated with autoimmunity or efficacy after alemtuzumab. Neurol Neuroimmunol Neuroinflamm..

[42] Hecker M, Fitzner B, Putscher E, Schwartz M, Winkelmann A, Meister S, Dudesek A, Koczan D, Lorenz P, Boxberger N, Zettl UK (2022). Implication of genetic variants in primary microRNA processing sites in the risk of multiple sclerosis. EBioMedicine.

[43] Putscher E, Hecker M, Fitzner B, Boxberger N, Schwartz M, Koczan D, Lorenz P, Zettl UK (2022). Genetic risk variants for multiple sclerosis are linked to differences in alternative pre-mRNA splicing. Front Immunol.

[44] Kurtzke JF (1983). Rating neurologic impairment in multiple sclerosis: an expanded disability status scale (EDSS). Neurology.

[45] Cossarizza A, Chang HD, Radbruch A, Abrignani S, Addo R, Akdis M, Andrä I, Andreata F, Annunziato F, Arranz E, Bacher P, Bari S, Barnaba V, Barros-Martins J, Baumjohann D, Beccaria CG, Bernardo D, Boardman DA, Borger J, Böttcher C, Brockmann L, Burns M, Busch DH, Cameron G, Cammarata I, Cassotta A, Chang Y, Chirdo FG, Christakou E, Čičin-Šain L, Cook L, Corbett AJ, Cornelis R, Cosmi L, Davey MS, De Biasi S, De Simone G, Del Zotto G, Delacher M, Di Rosa F (2021). Guidelines for the use of flow cytometry and cell sorting in immunological studies (third edition). Eur J Immunol..

[46] Morbach H, Eichhorn EM, Liese JG, Girschick HJ (2010). Reference values for B cell subpopulations from infancy to adulthood. Clin Exp Immunol.

[47] Thorarinsdottir K, Camponeschi A, Gjertsson I, Mårtensson IL (2015). CD21 -/low B cells: a snapshot of a unique B cell subset in health and disease. Scand J Immunol.

[48] Megyola C, Ye J, Bhaduri-McIntosh S (2011). Identification of a sub-population of B cells that proliferates after infection with Epstein–Barr virus. Virol J.

[49] Monaco G, Chen H, Poidinger M, Chen J, de Magalhães JP, Larbi A (2016). flowAI: automatic and interactive anomaly discerning tools for flow cytometry data. Bioinformatics.

[50] Bretz F, Hothorn T, Westfall P (2010). Multiple comparisons using R.

[51] Bates D, Mächler M, Bolker B, Walker S (2015). Fitting linear mixed-effects models using lme4. J Stat Softw.

[52] Fox J, Weisberg S (2018). An R companion to applied regression.

[53] Benjamini Y, Hochberg Y (1995). Controlling the false discovery rate: a practical and powerful approach to multiple testing. J R Stat Soc Series B Stat Methodol.

[54] Monaco G, Lee B, Xu W, Mustafah S, Hwang YY, Carré C, Burdin N, Visan L, Ceccarelli M, Poidinger M, Zippelius A, de Magalhães JP, Larbi A (2019). RNA-Seq signatures normalized by mRNA abundance allow absolute deconvolution of human immune cell types. Cell Rep.

[55] Falcon S, Gentleman R (2007). Using GOstats to test gene lists for GO term association. Bioinformatics.

[56] Safran M, Rosen N, Twik M, BarShir R, Stein TI, Dahary D, Fishilevich S, Lancet D. The GeneCards Suite. In: Abugessaisa I, Kasukawa T (eds). Practical guide to life science databases. Springer, Singapore. 2021. 10.1007/978-981-16-5812-9_2.

[57] Franz M, Rodriguez H, Lopes C, Zuberi K, Montojo J, Bader GD, Morris Q (2018). GeneMANIA update 2018. Nucleic Acids Res.

[58] Shannon P, Markiel A, Ozier O, Baliga NS, Wang JT, Ramage D, Amin N, Schwikowski B, Ideker T (2003). Cytoscape: a software environment for integrated models of biomolecular interaction networks. Genome Res.

[59] Sellner J, Rommer PS (2020). Immunological consequences of "immune reconstitution therapy" in multiple sclerosis: a systematic review. Autoimmun Rev..

[60] Kverneland AH, Streitz M, Geissler E, Hutchinson J, Vogt K, Boës D, Niemann N, Pedersen AE, Schlickeiser S, Sawitzki B (2016). Age and gender leucocytes variances and references values generated using the standardized ONE-Study protocol. Cytometry A.

[61] Oras A, Quirant-Sanchez B, Popadic D, Thunberg S, Winqvist O, Heck S, Cwikowski M, Riemann D, Seliger B, Martinez Caceres E, Uibo R, Giese T (2020). Comprehensive flow cytometric reference intervals of leukocyte subsets from six study centers across Europe. Clin Exp Immunol.

[62] Jalusic KO, Ellenberger D, Rommer P, Stahmann A, Zettl U, Berger K (2021). Effect of applying inclusion and exclusion criteria of phase III clinical trials to multiple sclerosis patients in routine clinical care. Mult Scler.

[63] Samjoo IA, Worthington E, Drudge C, Zhao M, Cameron C, Häring DA, Stoneman D, Klotz L, Adlard N (2021). Efficacy classification of modern therapies in multiple sclerosis. J Comp Eff Res.

[64] Ziemssen T, Bass AD, Berkovich R, Comi G, Eichau S, Hobart J, Hunter SF, LaGanke C, Limmroth V, Pelletier D, Pozzilli C, Schippling S, Sousa L, Traboulsee A, Uitdehaag BMJ, Van Wijmeersch B, Choudhry Z, Daizadeh N, Singer BA, CARE-MS I, CARE-MS II, CAMMS03409, and TOPAZ investigators (2020). Efficacy and safety of alemtuzumab through 9 years of follow-up in patients with highly active disease: post hoc analysis of CARE-MS I and II patients in the TOPAZ extension study. CNS Drugs.

[65] De Stefano N, Sormani MP, Giovannoni G, Rammohan K, Leist T, Coyle PK, Dangond F, Keller B, Alexandri N, Galazka A (2022). Analysis of frequency and severity of relapses in multiple sclerosis patients treated with cladribine tablets or placebo: the CLARITY and CLARITY extension studies. Mult Scler.

[66] Planas R, Jelčić I, Schippling S, Martin R, Sospedra M (2012). Natalizumab treatment perturbs memory- and marginal zone-like B-cell homing in secondary lymphoid organs in multiple sclerosis. Eur J Immunol.

[67] Traub JW, Pellkofer HL, Grondey K, Seeger I, Rowold C, Brück W, Husseini L, Häusser-Kinzel S, Weber MS (2019). Natalizumab promotes activation and pro-inflammatory differentiation of peripheral B cells in multiple sclerosis patients. J Neuroinflamm.

[68] Cuculiza Henriksen A, Ammitzbøll C, Petersen ER, McWilliam O, Sellebjerg F, von Essen MR, Romme Christensen J (2021). Natalizumab differentially affects plasmablasts and B cells in multiple sclerosis. Mult Scler Relat Disord..

[69] Gjertsson I, McGrath S, Grimstad K, Jonsson CA, Camponeschi A, Thorarinsdottir K, Mårtensson IL (2022). A close-up on the expanding landscape of CD21-/low B cells in humans. Clin Exp Immunol.

[70] Bar-Or A, Li R (2021). Cellular immunology of relapsing multiple sclerosis: interactions, checks, and balances. Lancet Neurol.

[71] Xu W, Seok J, Mindrinos MN, Schweitzer AC, Jiang H, Wilhelmy J, Clark TA, Kapur K, Xing Y, Faham M, Storey JD, Moldawer LL, Maier RV, Tompkins RG, Wong WH, Davis RW, Xiao W, Inflammation and Host Response to Injury Large-Scale Collaborative Research Program (2011). Human transcriptome array for high-throughput clinical studies. Proc Natl Acad Sci U S A..

[72] Nazarov PV, Muller A, Kaoma T, Nicot N, Maximo C, Birembaut P, Tran NL, Dittmar G, Vallar L (2017). RNA sequencing and transcriptome arrays analyses show opposing results for alternative splicing in patient derived samples. BMC Genomics.

[73] Erdei A, Kovács KG, Nagy-Baló Z, Lukácsi S, Mácsik-Valent B, Kurucz I, Bajtay Z (2021). New aspects in the regulation of human B cell functions by complement receptors CR1, CR2, CR3 and CR4. Immunol Lett.

[74] Verbeek JS, Hirose S, Nishimura H (2019). The complex association of FcγRIIb with autoimmune susceptibility. Front Immunol.

[75] Cotton RN, Wegrecki M, Cheng TY, Chen YL, Veerapen N, Le Nours J, Orgill DP, Pomahac B, Talbot SG, Willis R, Altman JD, de Jong A, Van Rhijn I, Clark RA, Besra GS, Ogg G, Rossjohn J, Moody DB (2021). CD1a selectively captures endogenous cellular lipids that broadly block T cell response. J Exp Med.

[76] Lim TS, Goh JK, Mortellaro A, Lim CT, Hämmerling GJ, Ricciardi-Castagnoli P (2012). CD80 and CD86 differentially regulate mechanical interactions of T-cells with antigen-presenting dendritic cells and B-cells. PLoS ONE.

[77] Marsh-Wakefield F, Juillard P, Ashhurst TM, Juillard A, Shinko D, Putri GH, Read MN, McGuire HM, Byrne SN, Hawke S, Grau GE (2022). Peripheral B-cell dysregulation is associated with relapse after long-term quiescence in patients with multiple sclerosis. Immunol Cell Biol.

[78] Walter MR (2014). The molecular basis of IL-10 function: from receptor structure to the onset of signaling. Curr Top Microbiol Immunol.

[79] Ghalamfarsa G, Mahmoudi M, Mohammadnia-Afrouzi M, Yazdani Y, Anvari E, Hadinia A, Ghanbari A, Setayesh M, Yousefi M, Jadidi-Niaragh F (2016). IL-21 and IL-21 receptor in the immunopathogenesis of multiple sclerosis. J Immunotoxicol.

[80] Jones JL, Phuah CL, Cox AL, Thompson SA, Ban M, Shawcross J, Walton A, Sawcer SJ, Compston A, Coles AJ (2009). IL-21 drives secondary autoimmunity in patients with multiple sclerosis, following therapeutic lymphocyte depletion with alemtuzumab (Campath-1H). J Clin Invest.

[81] Berchtold MW, Villalobo A (2014). The many faces of calmodulin in cell proliferation, programmed cell death, autophagy, and cancer. Biochim Biophys Acta.

[82] Yang Q, Tian S, Liu Z, Dong W (2021). Knockdown of RIPK2 inhibits proliferation and migration, and induces apoptosis via the NF-κB signaling pathway in gastric cancer. Front Genet.

[83] Zhang M, Liu J, Li M, Zhang S, Lu Y, Liang Y, Zhao K, Li Y (2018). Insulin-like growth factor 1/insulin-like growth factor 1 receptor signaling protects against cell apoptosis through the PI3K/AKT pathway in glioblastoma cells. Exp Ther Med.

[84] Zhang X, Yan C, Zhan X, Li L, Lei J, Shi Y (2018). Structure of the human activated spliceosome in three conformational states. Cell Res.

[85] Goldfarb KC, Cech TR (2017). Targeted CRISPR disruption reveals a role for RNase MRP RNA in human preribosomal RNA processing. Genes Dev.

[86] Jarrous N, Mani D, Ramanathan A (2022). Coordination of transcription and processing of tRNA. FEBS J.

[87] Bratkovič T, Božič J, Rogelj B (2020). Functional diversity of small nucleolar RNAs. Nucleic Acids Res.

[88] Pancho A, Aerts T, Mitsogiannis MD, Seuntjens E (2020). Protocadherins at the crossroad of signaling pathways. Front Mol Neurosci.

[89] Tamura N, Ohno K, Katayama T, Kanayama N, Sato K (2007). The PDZ-LIM protein CLP36 is required for actin stress fiber formation and focal adhesion assembly in BeWo cells. Biochem Biophys Res Commun.

[90] Fearnley GW, Young KA, Edgar JR, Antrobus R, Hay IM, Liang WC, Martinez-Martin N, Lin W, Deane JE, Sharpe HJ (2019). The homophilic receptor PTPRK selectively dephosphorylates multiple junctional regulators to promote cell-cell adhesion. Elife.

[91] Mal R, Magner A, David J, Datta J, Vallabhaneni M, Kassem M, Manouchehri J, Willingham N, Stover D, Vandeusen J, Sardesai S, Williams N, Wesolowski R, Lustberg M, Ganju RK, Ramaswamy B, Cherian MA (2020). Estrogen receptor beta (ERβ): a ligand activated tumor suppressor. Front Oncol.

[92] Mishra S, Murphy LC, Murphy LJ (2006). The Prohibitins: emerging roles in diverse functions. J Cell Mol Med.

[93] Lucas CR, Cordero-Nieves HM, Erbe RS, McAlees JW, Bhatia S, Hodes RJ, Campbell KS, Sanders VM (2013). Prohibitins and the cytoplasmic domain of CD86 cooperate to mediate CD86 signaling in B lymphocytes. J Immunol.

[94] Jiang T, Wang J, Li C, Cao G, Wang X (2022). Prohibitins: a key link between mitochondria and nervous system diseases. Oxid Med Cell Longev.

[95] Tan D, Zhou M, Kiledjian M, Tong L (2014). The ROQ domain of Roquin recognizes mRNA constitutive-decay element and double-stranded RNA. Nat Struct Mol Biol.

[96] Srivastava M, Duan G, Kershaw NJ, Athanasopoulos V, Yeo JH, Ose T, Hu D, Brown SH, Jergic S, Patel HR, Pratama A, Richards S, Verma A, Jones EY, Heissmeyer V, Preiss T, Dixon NE, Chong MM, Babon JJ, Vinuesa CG (2015). Roquin binds microRNA-146a and Argonaute2 to regulate microRNA homeostasis. Nat Commun.

[97] Moser T, Hoepner L, Schwenker K, Seiberl M, Feige J, Akgün K, Haschke-Becher E, Ziemssen T, Sellner J (2021). Cladribine alters immune cell surface molecules for adhesion and costimulation: further insights to the mode of action in multiple sclerosis. Cells.

[98] Green DR (2022). The mitochondrial pathway of apoptosis part II: The BCL-2 protein family. Cold Spring Harb Perspect Biol..

[99] Tischner D, Woess C, Ottina E, Villunger A (2010). Bcl-2-regulated cell death signalling in the prevention of autoimmunity. Cell Death Dis.

[100] Bardwell PD, Gu J, McCarthy D, Wallace C, Bryant S, Goess C, Mathieu S, Grinnell C, Erickson J, Rosenberg SH, Schwartz AJ, Hugunin M, Tarcsa E, Elmore SW, McRae B, Murtaza A, Wang LC, Ghayur T (2009). The Bcl-2 family antagonist ABT-737 significantly inhibits multiple animal models of autoimmunity. J Immunol.

[101] Junttila IS (2018). Tuning the cytokine responses: an update on interleukin (IL)-4 and IL-13 receptor complexes. Front Immunol.

[102] Ochi H, Osoegawa M, Wu XM, Minohara M, Horiuchi I, Murai H, Furuya H, Kira J (2002). Increased IL-13 but not IL-5 production by CD4-positive T cells and CD8-positive T cells in multiple sclerosis during relapse phase. J Neurol Sci.

[103] Ghezzi L, Cantoni C, Cignarella F, Bollman B, Cross AH, Salter A, Galimberti D, Cella M, Piccio L (2020). T cells producing GM-CSF and IL-13 are enriched in the cerebrospinal fluid of relapsing MS patients. Mult Scler.

[104] Bröer S (2014). The SLC38 family of sodium-amino acid co-transporters. Pflugers Arch.

[105] Aggarwal T, Patil S, Ceder M, Hayder M, Fredriksson R (2020). Knockdown of SLC38 transporter ortholog—CG13743 reveals a metabolic relevance in drosophila. Front Physiol.

[106] Pfeuffer S, Ruck T, Pul R, Rolfes L, Korsukewitz C, Pawlitzki M, Wildemann B, Klotz L, Kleinschnitz C, Scalfari A, Wiendl H, Meuth SG (2021). Impact of previous disease-modifying treatment on effectiveness and safety outcomes, among patients with multiple sclerosis treated with alemtuzumab. J Neurol Neurosurg Psychiatry.

[107] Lee J, Hyeon DY, Hwang D (2020). Single-cell multiomics: technologies and data analysis methods. Exp Mol Med.

[108] Ruck T, Barman S, Schulte-Mecklenbeck A, Pfeuffer S, Steffen F, Nelke C, Schroeter CB, Willison A, Heming M, Müntefering T, Melzer N, Krämer J, Lindner M, Riepenhausen M, Gross CC, Klotz L, Bittner S, Muraro PA, Schneider-Hohendorf T, Schwab N, Meyer Zu Hörste G, Goebels N, Meuth SG, Wiendl H (2022). Alemtuzumab-induced immune phenotype and repertoire changes: implications for secondary autoimmunity. Brain.

[109] Ruschil C, Gabernet G, Kemmerer CL, Jarboui MA, Klose F, Poli S, Ziemann U, Nahnsen S, Kowarik MC (2023). Cladribine treatment specifically affects peripheral blood memory B cell clones and clonal expansion in multiple sclerosis patients. Front Immunol.

