# Supplementary material for: Transcriptome alterations in peripheral blood B cells of patients with multiple sclerosis receiving immune reconstitution therapy
Source: J Neuroinflammation. 2023 Aug 2;20:181. doi: 10.1186/s12974-023-02859-x (PMC10394872; doi:10.1186/s12974-023-02859-x)
Supplement: Supplementary file 1 — Additional file 1. Gating strategy for the B-cell phenotyping as well as cell population shifts and expression dynamics during alemtuzumab therapy. [file 12974_2023_2859_MOESM1_ESM.pdf]

Additional file 1 for

Transcriptome alterations in peripheral blood B cells of patients with multiple sclerosis receiving immune reconstitution therapy

Michael Hecker[1,*], Brit Fitzner[1], Nina Boxberger[1], Elena Putscher[1], Robby Engelmann[2], Wendy Bergmann[3], Michael Müller[3], Isis Ludwig-Portugall[4], Margit Schwartz[1], Stefanie Meister[1], Ales Dudesek[1], Alexander Winkelmann[1], Dirk Koczan[5], Uwe Klaus Zettl[1]

[1] Rostock University Medical Center, Department of Neurology, Division of Neuroimmunology, Gehlsheimer Str. 20, 18147 Rostock, Germany

[2] Rostock University Medical Center, Clinic III (Hematology, Oncology and Palliative Medicine), Special Hematology Laboratory, Ernst-Heydemann-Str. 6, 18057 Rostock, Germany

[3] Rostock University Medical Center, Core Facility for Cell Sorting and Cell Analysis, Schillingallee 70, 18057 Rostock, Germany

[4] Miltenyi Biotec B.V. & Co. KG, Robert-Koch-Str. 1, 17166 Teterow, Germany

[5] Rostock University Medical Center, Institute of Immunology, Schillingallee 70, 18057 Rostock, Germany

[*] corresponding author: Michael Hecker, e-mail: michael.hecker@rocketmail.com, ORCID ID: 0000-0001-7015-3094

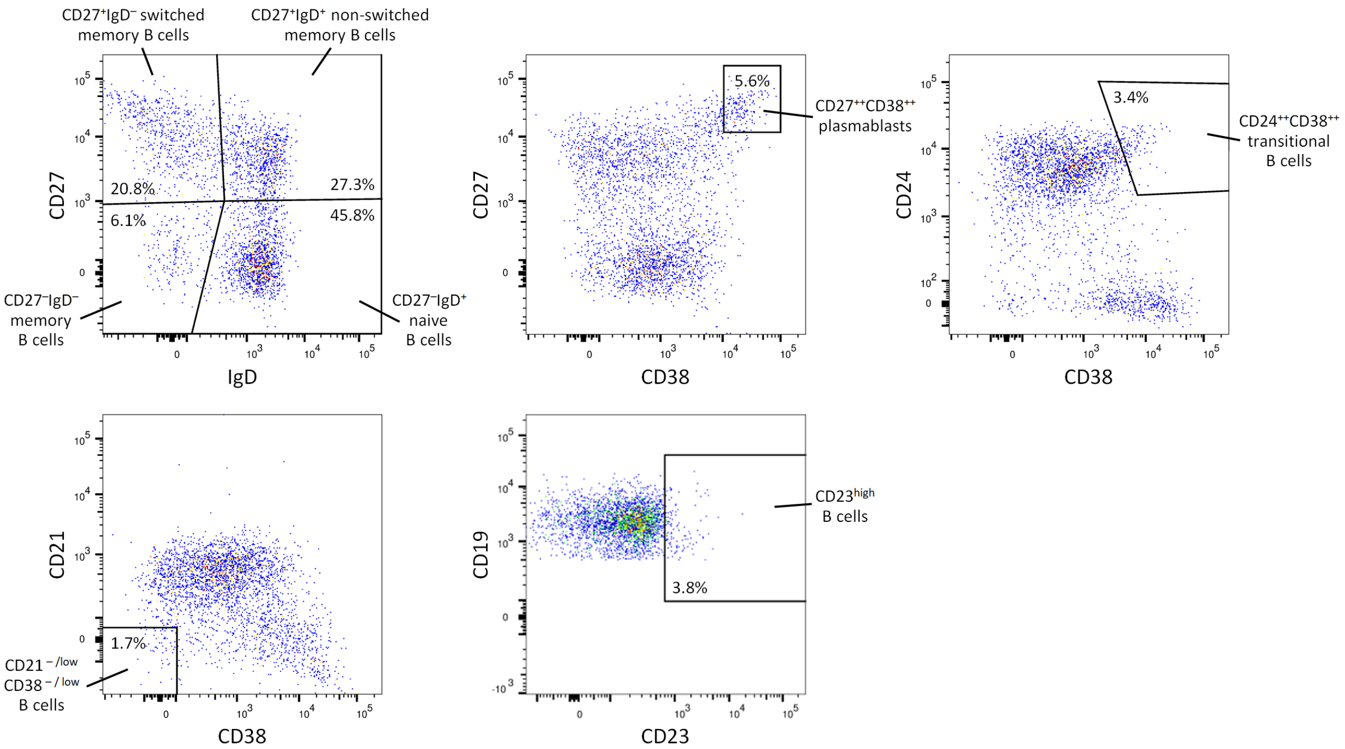

Fig. S1 : Gating strategy for the B-cell phenotyping.

Shown are exemplary data obtained by flow cytometry of thawed cryopreserved cells of a healthy subject. The plots visualize all events that resulted from the gating of live single CD19^{+} cells. Eight B-cell subpopulations were identified by the markers CD21, CD23, CD24, CD27, CD38 and IgD following the recommendations by Morbach et al. (Clin Exp Immunol, 2010) and Cossarizza et al. (Eur J Immunol, 2021). CD27 was plotted against IgD to identify CD27^{+}IgD^{-} switched memory B cells, CD27^{+}IgD^{+} non-switched memory B cells, CD27^{-}IgD^{-} memory B cells and CD27^{-}IgD^{+} naive B cells. Plasmablasts were defined as CD27^{++}CD38^{++} cells and transitional B cells as CD24^{++}CD38^{++} cells. Additionally, CD21$^{-/low}$CD38$^{-/low}$ B cells (Isnardi et al., Blood, 2010) and CD23^{high} B cells (Bonnefoy et al., Curr Opin Immunol, 1995) were gated. Percentages in the gates are given relative to all live single CD19^{+} cells.

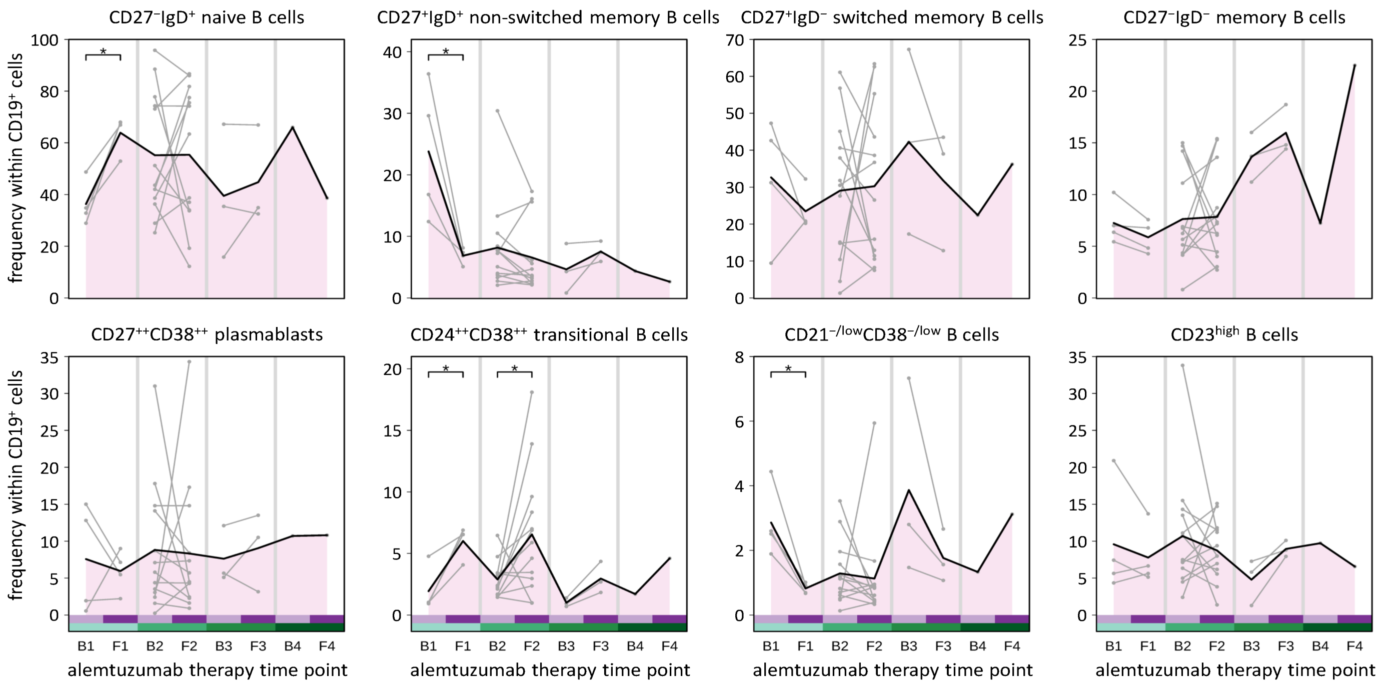

Fig. S2 : Frequencies of B-cell subsets across alemtuzumab therapy time points.

Area charts showing the average frequencies of different B-cell subpopulations before (B) and following (F) the 1st, 2nd, 3rd and 4th alemtuzumab treatment course. The superimposed gray lines connect the data of paired samples. The brackets indicate statistical significance in paired *t* tests. After the start of alemtuzumab therapy, there was a significant increase in the proportions of $CD27^{-}IgD^{+}$ naive B cells and $CD24^{++}CD38^{++}$ transitional B cells and a significant decrease in the proportions of $CD27^{+}IgD^{+}$ non-switched memory B cells and $CD21^{-/low}CD38^{-/low}$ B cells. * $p<0.05$.

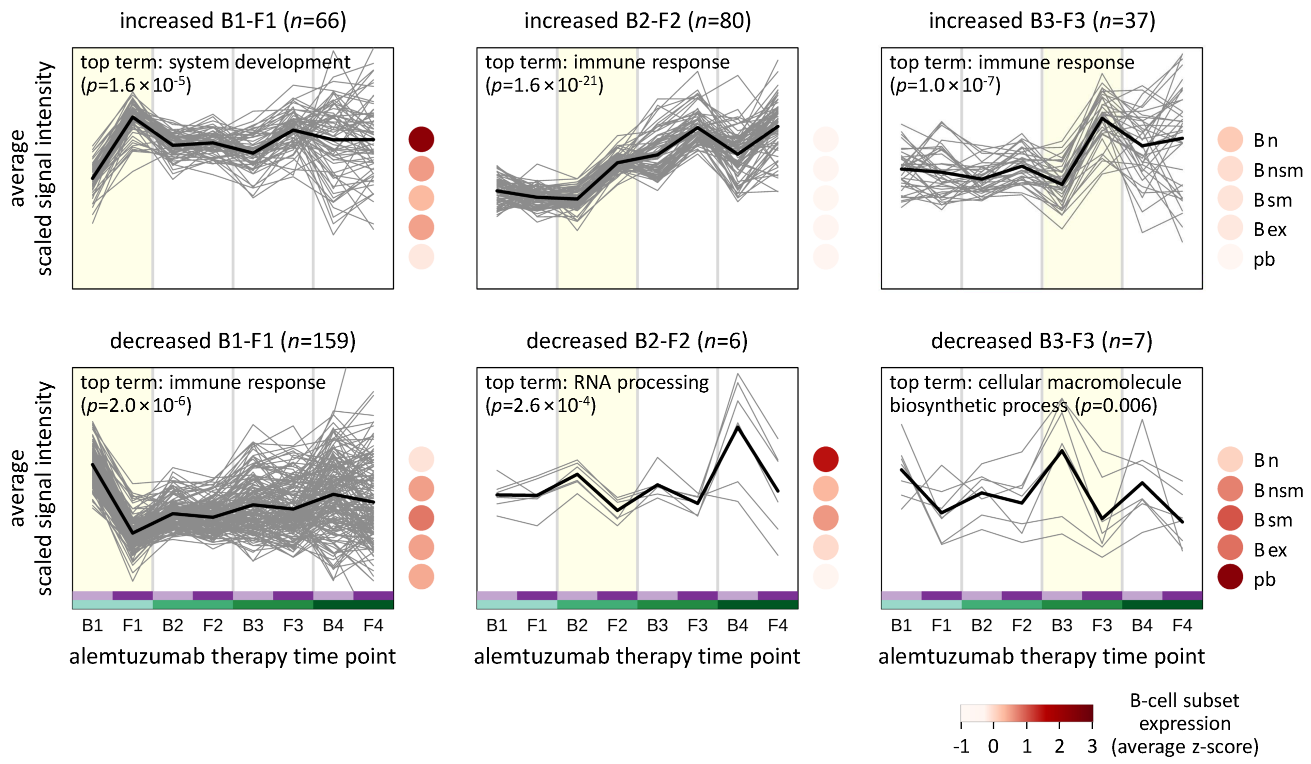

Fig. S3 : Genes with significantly altered expression in response to alemtuzumab.

B-cell transcriptome profiles were compared before (B) and following (F) the first, second and third treatment course. The analysis of the paired samples revealed genes with significantly increased or decreased expression, which are shown in the top and bottom subpanels, respectively. A line is drawn for each differentially expressed gene by connecting the average standardized expression value per time point. The thick line in each subpanel shows the mean of the means over all genes. The most overrepresented gene functional term that is characteristic of the respective gene set is given in the upper left corner. In addition, the dot plots on the right of each subpanel visualize the average expression of the genes in different B-cell subsets according to the dataset by Monaco et al. (Cell Rep, 2019). B ex = exhausted memory B cells, B n = naive B cells, B nsm = non-switched memory B cells, B sm = switched memory B cells, pb = plasmablasts.

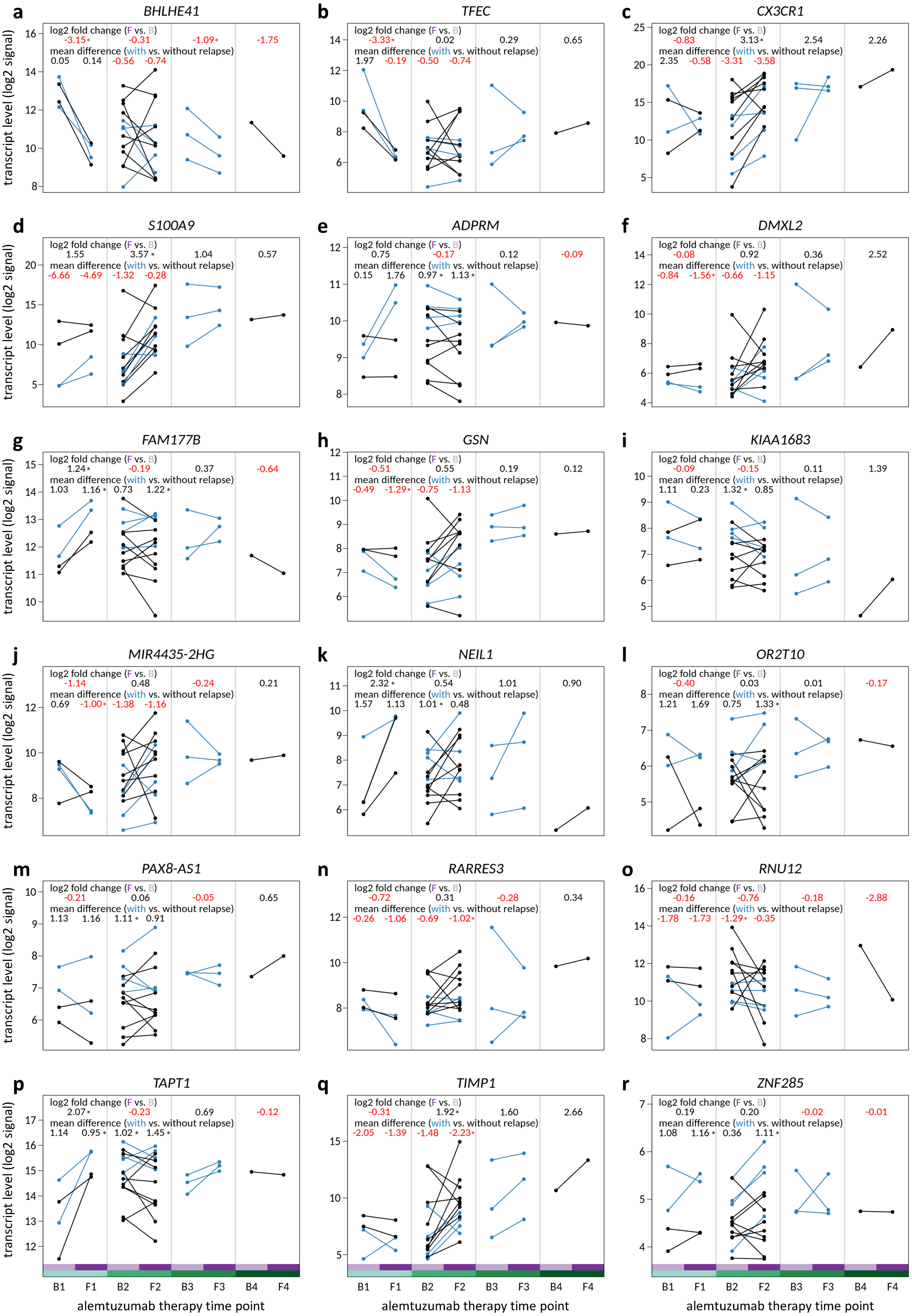

a BHLHE41
b TFEC
c CX3CR1
d S100A9
e ADPRM
f DMXL2
g FAM177B
h GSN
i KIAA1683
j MIR4435-2HG
k NEIL1
l OR2T10
m PAX8-AS1
n RARRES3
o RNU12
p TAPT1
q TIMP1
r ZNF285
log2 fold change (F vs. B)
mean difference (with vs. without relapse)
transcript level (log2 signal)
B1 F1 B2 F2 B3 F3 B4 F4
alemtuzumab therapy time point

Fig. S4 : Expression dynamics during alemtuzumab therapy (supplement to Figure 6).

For 18 genes, the transcript levels in B cells before (B) as well as following (F) an alemtuzumab treatment course are shown. Lines connect the data for the B sample and the F sample from the same patient (*n*=21 sample pairs). Blue dots/lines indicate that the patient experienced a relapse in the 12 months following drug administration (*n*=2 and *n*=4 for the first and second treatment course, respectively) while black dots/lines indicate that the patient was free of relapses in the follow-up. (**a**,**b**) Extremely lower expression levels (log2 fold change <−3 and *p*<0.05) were seen for *BHLHE41* and *TFEC* following the start of alemtuzumab therapy. (**c**,**d**) Extremely higher expression levels (log2 fold change >3 and *p*<0.05) were seen for *CX3CR1* and *S100A9* after the second treatment course. (**e**-**r**) The other genes were significantly differentially expressed between patients with and without relapse (mean difference <−1 or >1 at B1 and B2 or at F1 and F2 and *p*<0.05 at one time point). Negative values are displayed in red. * *p*<0.05.
